# Thyroid hormone remodels cortex to coordinate body-wide metabolism and exploration

**DOI:** 10.1016/j.cell.2024.07.041

**Published:** 2024-08-22

**Authors:** Daniel R. Hochbaum, Lauren Hulshof, Amanda Urke, Wengang Wang, Alexandra C. Dubinsky, Hannah C. Farnsworth, Richard Hakim, Sherry Lin, Giona Kleinberg, Keiramarie Robertson, Canaria Park, Alyssa Solberg, Yechan Yang, Caroline Baynard, Naeem M. Nadaf, Celia C. Beron, Allison E. Girasole, Lynne Chantranupong, Marissa D. Cortopassi, Shannon Prouty, Ludwig Geistlinger, Alexander S. Banks, Thomas S. Scanlan, Sandeep Robert Datta, Michael E. Greenberg, Gabriella L. Boulting, Evan Z. Macosko, Bernardo L. Sabatini

**Affiliations:** 1 Howard Hughes Medical Institute, Department of Neurobiology, Harvard Medical School, Boston, MA 02115, USA; 2 Society of Fellows, Harvard University, Cambridge, MA 02138, USA; 3 Broad Institute of MIT and Harvard, Cambridge, MA 02142, USA; 4 Department of Biomedical Informatics, Harvard Medical School, Boston, MA 02115, USA; 5 Department of Neurobiology, Harvard Medical School, Boston, MA 02115, USA; 6 Division of Endocrinology, Diabetes, and Metabolism, Beth Israel Deaconess Medical Center and Harvard Medical School, Boston, MA 02215, USA; 7 Department of Neurobiology, University of Massachusetts Chan Medical School, Worcester, MA 01655, USA; 8 Center for Computational Biomedicine, Harvard Medical School, Boston, MA 02215, USA; 9 Department of Chemical Physiology & Biochemistry, Oregon Health & Science University, Portland, OR 97239, USA; 10 Department of Psychiatry, Massachusetts General Hospital, Boston, MA 02114, USA; 11 These authors contributed equally; 12 Lead contact

## Abstract

Animals adapt to environmental conditions by modifying the function of their internal organs, including the brain. To be adaptive, alterations in behavior must be coordinated with the functional state of organs throughout the body. Here, we find that thyroid hormone—a regulator of metabolism in many peripheral organs—directly activates cell-type-specific transcriptional programs in the frontal cortex of adult male mice. These programs are enriched for axon-guidance genes in glutamatergic projection neurons, synaptic regulatory genes in both astrocytes and neurons, and pro-myelination factors in oligodendrocytes, suggesting widespread plasticity of cortical circuits. Indeed, whole-cell electrophysiology revealed that thyroid hormone alters excitatory and inhibitory synaptic transmission, an effect that requires thyroid hormone-induced gene regulatory programs in presynaptic neurons. Furthermore, thyroid hormone action in the frontal cortex regulates innate exploratory behaviors and causally promotes exploratory decision-making. Thus, thyroid hormone acts directly on the cerebral cortex in males to coordinate exploratory behaviors with whole-body metabolic state.

## In brief

The direct action of thyroid hormone on the cortical circuits of male mice integrates exploratory behaviors with whole-body metabolism.

## Graphical Abstract

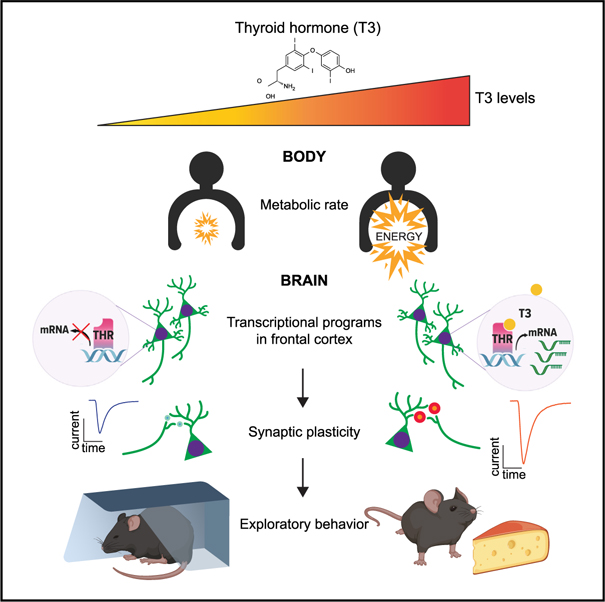

## INTRODUCTION

In response to varying environmental conditions, such as seasonal food availability and weather, animals coordinate alterations in the function of their organs with changes in behavior. For example, snakes that undergo long periods of fasting will, after swallowing their prey, reassemble digestive organs and seek atypical habitats in which they become largely immobile.^1^ Similarly, animals that hibernate drastically change organ function, metabolism, and body temperature while also engaging a dormant behavioral state with suppressed thirst and feeding drives.^2^ The coordination of peripheral organ function with changes in behavior is likely adaptive, enabling animals to survive in fluctuating environments.

The signals that coordinate these processes include circulating hormones that are produced in and regulate peripheral organs but also enter the brain, where they act on receptors expressed by diverse classes of cells. Hormones are often components of homeostatic control systems mediated by the hypothalamus—e.g., leptin produced by adipocytes acts via hypothalamic circuits to reduce food consumption and increase energy expenditure, thus stabilizing body fat composition.^3,4^ However, circulating hormones have additional effects in the brain that can modulate behavior and cognition. For instance, leptin influences visual cortices to control the degree to which brain cells consume energy to code visual information, triggering expenditure of cellular energy supplies, when abundant, to improve visual perception.^5^ Similarly, female sex hormones essential for ovarian and menstrual cycles as well as fetal development enter the brain and induce nest building and other behaviors that must be coordinated with pregnancy.^6,7^

Thyroid hormone, which is produced in the thyroid gland and acts on many tissues to regulate metabolism and function, affects both the periphery and the brain. Its active form, triiodothyronine (T3), stimulates lipolysis and fatty acid metabolism in the liver,^8^ kindles thermogenesis in adipose tissue,^9^ and increases energy expenditure, fast contractility, and glycolysis in skeletal muscles,^10,11^ but also enters the brain and acts via the hypothalamic-pituitary-thyroid (HPT) axis to inhibit its own production and stabilize its circulating levels.^12,13^ T3 also acts on hypothalamic neurons to regulate body temperature, food intake, and weight^14,15^ (among other functions^16,17^). These observations in animals are consistent with changes in body temperature and weight in humans with pathologically low or high thyroid levels characteristic of hypo- or hyper-thyroidism, respectively.^18,19^

Seasonal fluctuations in thyroid hormone levels are pronounced in many mammalian species.^20^ For instance, thyroid hormone levels surge in Madagascar gray mouse lemurs during their resource-abundant wet season. As a result, these primates upregulate their metabolism, increasing caloric intake 4-fold and oxygen consumption 2-fold.^21^ These animals also undergo dramatic sex-dependent changes in behavior: males expand their home territory in synchrony with increasing thyroid hormone levels, spending more time awake, foraging for food, and searching for mates.^22,23^ These exploratory behaviors leave male lemurs more prone to predation, and as a result, their mortality rate increases during this season.^24^ Similar correlations between thyroid levels and exploratory-like behaviors have been observed in many species.^17,25–28^ These observations, and others,^29^ implicate sexually dimorphic effects of thyroid hormone on exploratory behaviors.

Further evidence that thyroid hormone regulates exploratory behaviors comes from humans with thyroid dysfunction. Individuals with hypo- or hyper-thyroidism often exhibit psychiatric symptoms, including depression or mania, respectively.^30–33^ These symptoms may represent pathological extremes of normal variation in exploratory behavior, with mania characterized as an over-expression of exploration relative to risks,^34,35^ and depression as a neglect of exploratory behaviors despite potential gains and absence of risks.^36,37^ Thyroid dysregulation in humans is more prevalent in females as sex-specific immune function increases their incidence of autoimmune thyroid disease.^38,39^ Few studies examined sex-specific behaviors in human thyroid disorders^40^; however, associations between non-pathological thyroid hormone levels and socioeconomic outcomes, such as employment status and household income, differ markedly between males and females.^41^ Collectively, evidence from animals and humans suggests that thyroid hormone controls both metabolic state and exploratory behaviors, potentially in a sex-dependent manner.

The effects of thyroid hormone are mediated by thyroid hormone receptors (THRs), ligand-gated transcription factors that bind T3 with high affinity.^12^ Unlike many steroid hormone receptors that translocate to the nucleus upon ligand binding, unliganded THRs bind to DNA and recruit co-repressors. T3 binding to THRs causes remodeling of chromatin, resulting in the dissociation of co-repressors and recruitment of transcriptional activators.^42^ Thus, THRs repress gene transcription in the absence of T3 and activate transcription in the presence of T3. Furthermore, germline deletion or mutation of THRs in mice dysregulates the HPT axis and induces hyperactive behavioral phenotypes,^43,44^ suggesting a direct influence of thyroid-dependent gene expression on behavior. THRs are abundant across cerebral cortex in rodents^45,46^ and humans.^47^ Although thyroid signaling is critical for proper cortical development,^48,49^ its function in the adult cortex is unknown. Frontal cortical structures such as secondary motor cortex (M2), which influence both goal-oriented and exploratory actions and decision-making, express THRs. Here, we find that T3 induces plasticity of frontal cortical circuits in male mice through engagement of local thyroid-dependent transcriptional programs to coordinate exploratory-like behaviors with changes in body-wide metabolic state.

## RESULTS

### T3-modulated metabolism and behavior

We hypothesized that T3 directly affects cortical brain areas, such as M2, that express THRs.^45,46^ Therefore, we developed a T3-delivery paradigm that induces transcriptional changes in M2 of C57BL/6J mice and examined the utility of these mice for detecting T3-induced behavioral changes.

**Figure 1.**
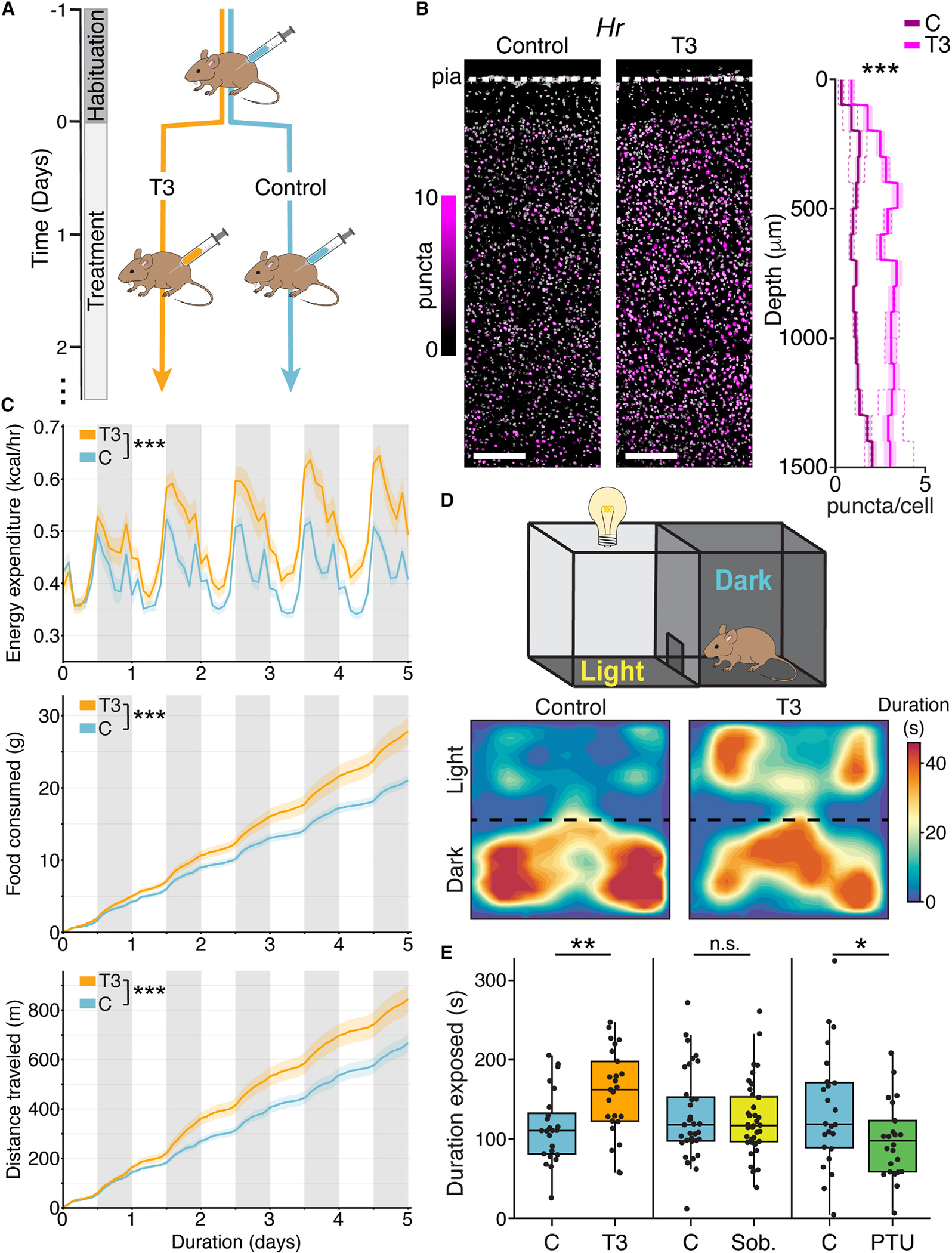
Brain-penetrant T3 induces transcription in cortex and modulates spontaneous exploratory behaviors (A) Mice were habituated to IP injections with vehicle and were then divided into T3 and vehicle control cohorts. (B) FISH of *Hr* in M2 (left: control; middle: T3; nuclei pseudo-colored by number of puncta, see Figure S1E). Scale bars, 200 μm. Right: *Hr* expression as a function of cortical depth. *Hr* is upregulated by T3 across cortex (*p* = 0, Wilcoxon rank-sum test comparing treatment effect across entire cortical depth; control: *n* = 5,494 cells; T3: *n* = 6,061; 2 mice per condition). Central line/shade: mean/95% confidence intervals. Dotted lines: mean expression values per mouse. (C) Home-cage indirect calorimetry revealed that energy expenditure, food consumption, and distance traveled significantly increased with T3 treatment (*p* < 10^−4^, likelihood ratio tests, *n* = 16 control, *n* = 15 T3-treated mice). Central line/shade: mean/SEM. (D) Top: schematic of light-dark preference assay (LD). Bottom: example heatmaps of the duration that a control (left) and a T3-treated (right) mouse occupied each area of the LD box. (E) Duration male mice stayed in the light-exposed zone increased with T3 (left) (*p* = 0.004, *n* = 24 control, *n* = 25 T3 mice), was unaffected by sobetirome (middle) (*p* = 0.818, *n* = 35 control, *n* = 36 sobetirome mice), and decreased with PTU (right) (*p* = 0.04, *n* = 24 control, *n* = 24 mice). Welch’s t tests. Central line: median, box: IQ, whiskers: data within 1.5× IQR. Black dots indicate data from single mice. For all panels: n.s., not significant; *p < 0.05, **p < 0.01, ***p < 0.001. See also Figures S1, S2, and S3.

T3 was administered to adult mice, and comparisons were made to animals treated with vehicle alone (Figure 1A). We used the expression of genes previously identified as responsive to T3 in primary neocortical cultures^50^ (*Hr*, *Ier5*, *Cyp11a1*) as a readout of T3 entrance into, and action on, the brain. Expression of thyroid-responsive genes (TRGs) in frontal cortex increased with levels of administered T3 up to ~0.1 μg/g (Figures S1A–S1C) and was observed within 1 h of treatment (Figure S1D). Fluorescence *in situ* hybridization (FISH) of *Hr* and *Ier5* demonstrated widespread induction by T3 expression in M2 (Figures 1B, S1E, and S1F), consistent with the induction of transcriptional programs in adult cortex by brain-penetrant T3.

Home-cage indirect calorimetry revealed T3-induced changes to physiological processes consistent with a hyperthyroid state. T3 administration increased energy expenditure, body temperature, food intake, and locomotion over the experimental time course (Figures 1C and S1G). T3 treatment increased the likelihood that animals were active without increasing locomotion within each activity bout (Figures S1H and S1I).

To examine if increased thyroid hormone levels promote exploratory-like behaviors, we used a light-dark (LD) preference assay in which mice were placed in a box that contained both dark and lit areas. Mice naturally find the light-exposed region aversive and spend most of their time in the sheltered, dark region. T3 treatment (3.5 days) had no effects on locomotion in either male or female mice (Figures S2A and S2B). However, male mice treated with T3 spent significantly more time exploring the light-exposed area than vehicle-treated males (Figures 1D and 1E). By contrast, T3-treated female mice showed no significant increase in time spent in the light-exposed area compared with vehicle-treated females (Figure S2C). These results are consistent with studies implicating thyroid hormone in sexually dimorphic behaviors in many species^17,20–29^ and motivated our further analyses of male mice.

The magnitude of the effect on male mice in the LD assay increased with T3 concentration and remained significantly elevated even at the lowest levels tested (Figure S2D; 0.016 μg/g). As T3 affects many organs that might secondarily impact the brain and behavior, we examined if T3 entry into the brain is necessary to alter behavior in the LD assay. Treatment with sobetirome (0.1 μg/g), a T3-mimetic with poor blood-brain barrier permeability,^51,52^ induced TRGs peripherally but not centrally and increased energy expenditure similar to T3 (Figures S2E–S2G). However, sobetirome did not alter the duration mice spent in the exposed area (Figure 1E). Therefore, peripheral actions of thyroid hormone are not sufficient to alter behavior in the LD assay. Finally, chronic treatment (3.5 weeks) with propylthiouracil (PTU), which interferes with thyroid hormone synthesis and gradually reduces T3,^53^ reduced the time spent in the illuminated zone and repressed TRGs in cortex (Figures 1E and S2H). Collectively, these data are consistent with T3 acting directly in the brain to bidirectionally regulate exploratory behaviors.

The behavioral effects of T3 administration were not due to general hyperactivity as locomotion was unaffected during the LD assay, in an open-field (Figure S2I), or during motion sequencing (MoSeq) analysis of spontaneous behavior. The latter is a machine learning approach^54^ that parses animal movement into a sequence of repeated “syllables” with stereotyped postural dynamics (Figure S3A). MoSeq confirmed that increasing T3 did not affect locomotion, velocity, or position within the arena; however, T3 made animals less predictable in syllable usage and syllable sequences, resulting in more diverse behavioral patterns consistent with increased spontaneous behavioral exploration (Figures S3B–S3I).

### T3 induces distinct circuit remodeling programs across cell types

**Figure 2.**
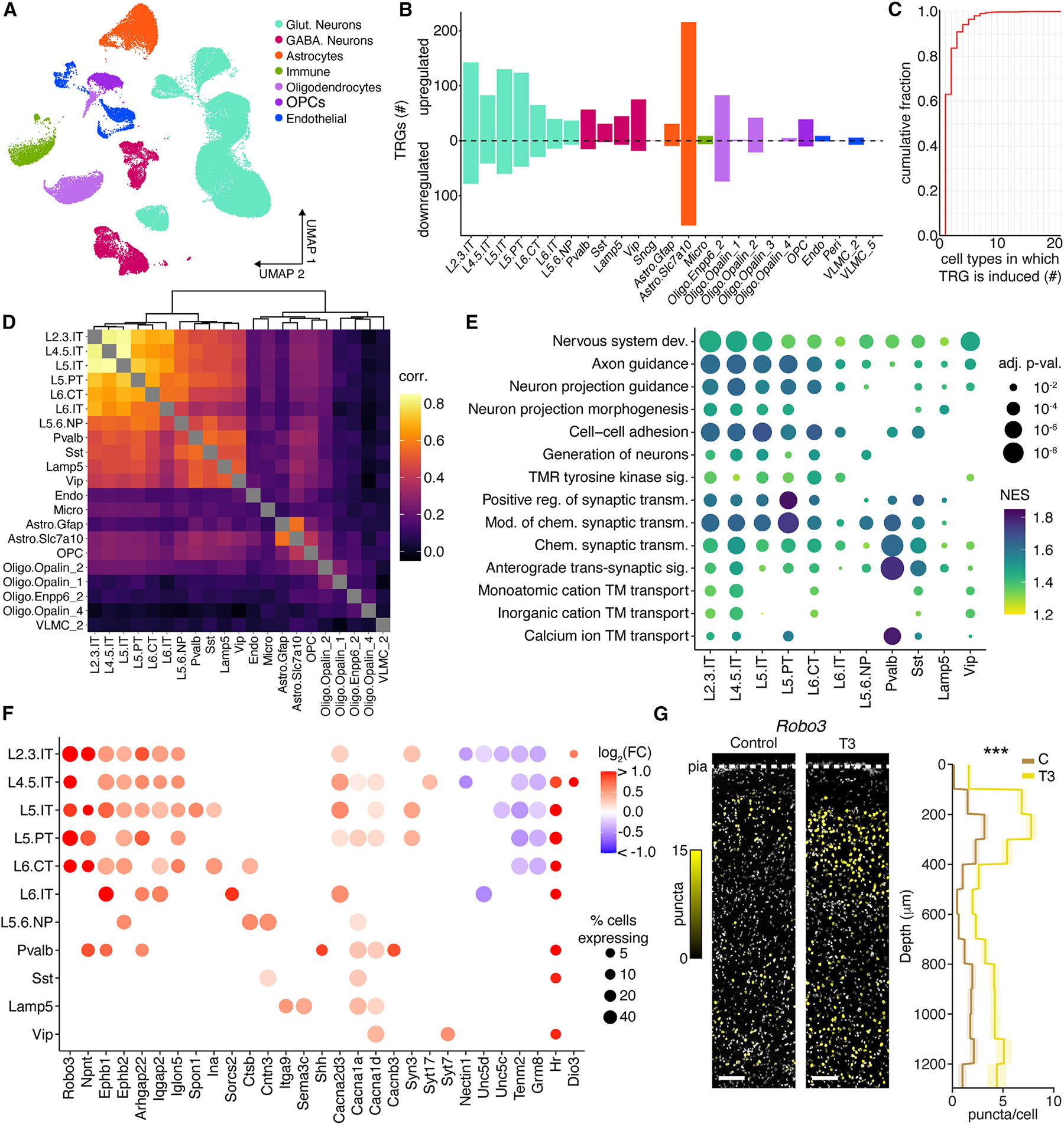
snRNA-seq of M2 reveals cell-type-specific T3-induced transcriptional programs (A) Uniform manifold approximation and projection (UMAP) representation of nuclear transcriptomes (each dot = one nucleus). Sequenced nuclei clustered into broad cell classes and were subsequently mapped onto specific cell types (Figure S4B). (B) The numbers of TRGs within each cell type (false discovery rate [FDR]-adjusted *p* < 0.05, |robScore_logFC| ≥ 0.5). Full list of TRGs: Table S1. (C) Cumulative distribution of the number of cell types in which TRGs are induced. Most TRGs (~60%) were detected in only one cell type. (D) Heatmap representation of the Spearman correlation of changes in expression of TRGs across cell types. Cell types without TRGs were excluded. The dendrogram shows the hierarchical clustering of cell types based on changes in expression of TRGs. (E) Dot plot of top terms from GSEA of TRGs in glutamatergic and GABAergic neurons. The union of the top 3 terms per cell type is displayed. Color: normalized enrichment score for a given term associated with TRGs. Size: FDR-adjusted *p* value. TM, transmembrane; TMR, transmembrane receptor. Full GSEA results for each cell type with sufficient sample size: Table S2. (F) Dot plot of TRGs expression changes across glutamatergic and GABAergic neurons. Neuronal TRGs were enriched for many genes driving axon-guidance GSEA terms, including *Robo3*, *Npnt*, and ephrins (*Ephb1*, *Ephb2*) in glutamatergic projection neurons, and genes associated with presynaptic function such as *Cacna2d3*, *Cacna1a*, *Syn3*, and synaptotagmins (*Syt7*, *Syt17*). Downregulated genes included *Grm8*, a presynaptic, putative negative regulator of glutamatergic transmission.^56^ Color: fold-change in expression level. Size: percent of cells expressing the TRG in the T3 state. (G) FISH of *Robo3* in M2 (left: control; middle: T3; nuclei pseudo-colored by number of puncta, see Figure S4D). Scale bars, 100 μm. Right: *Robo3* expression as a function of depth. *Robo3* is upregulated by T3 across cortical layers (*p* < 10^−4^, Wilcoxon rank-sum test comparing treatment effect across entire cortical depth; control: *n* = 9,675 cells; T3: *n* = 16,105; 3 mice per condition). Central line/shade: mean/95% confidence intervals. See also Figure S4.

To identify transcriptional programs that may mediate thyroid-dependent changes in exploratory behaviors, we conducted single-nucleus RNA sequencing (snRNA-seq) of dorsal frontal cortex, centered on M2, of control mice or those exposed to T3 for 2.5 days (*n* = 4/condition). After quality control, we analyzed a dataset of 52,996 cells from T3-treated and 54,746 cells from control animals, which were assigned cell type labels using a recent single-cell motor cortex reference^55^ (Figures 2A, S4A, and S4B).

For each cell type, we identified differentially expressed TRGs between control and elevated T3 conditions (Table S1). T3 altered gene expression across most cell types with 699 and 414 unique statistically significant up- and downregulated genes, respectively (Figure 2B). TRGs were largely cell-type-specific, with only 16% differentially expressed in three or more types (Figure 2C). Similarly, hierarchical clustering revealed distinct TRG programs within neuronal and non-neuronal cell types (Figure 2D).

Astrocytes are a major source of brain T3 through active transport of the pro-hormone thyroxine (T4) across the blood-brain barrier, and conversion of T4 to T3 by the type 2 deiodinase, *Dio2*. Furthermore, T3 regulates astrocyte differentiation, morphogenesis, and maturation.^57^ We find that, in response to T3, astrocytes downregulate both *Slco1a1*, the major astrocytic transporter of T4, and *Dio2*, revealing a homeostatic mechanism to stabilize T3 levels by reducing T4 intake and conversion to T3 (Figure S4C). Gene set enrichment analysis (GSEA) of astrocyte TRGs (Table S2) revealed an over-representation of genes associated with synapse formation and maintenance, including astrocyte-expressed genes that regulate synaptic glutamate release and clearance (Figure S4C). Thus, T3 induces an astrocyte-specific transcriptional program that may modulate glutamatergic synapses.

Oligodendrocyte progenitor cell (OPC) differentiation and oligodendrocyte maturation are critically dependent on T3,^58–61^ and T3 mimetics are being explored as treatments for adult demyelination disorders.^62^ Oligodendrocytes and OPCs responded to T3 with 246 TRGs enriched for regulators of OPC differentiation and oligodendrocyte myelination and with different programs across OPCs, immature (Oligo.Enpp6_2), and mature oligodendrocytes (Figures 2B, 2D, and S4C). Therefore, in response to T3, adult oligodendrocytes and OPCs induce maturation stage-specific genetic programs that likely regulate differentiation, structural remodeling, and myelination.

Within glutamatergic and GABAergic neurons, the top T3-regulated pathways revealed a striking enrichment for genes associated with axon guidance and plasticity in projecting glutamatergic neurons and synaptic regulation across both glutamatergic neurons and subtypes of GABAergic neurons (Figure 2E). TRGs included many implicated in axon pathfinding, axonal and presynaptic localized cell-cell adhesion, calcium handling, and other presynaptic functions (Figure 2F). Among these, *Robo3* stood out as highly induced across most glutamatergic cell types (~2- to 4-fold increase). T3-induced increases in *Robo3* transcripts across layers of M2 were confirmed by FISH and in Robo3 protein by western blotting (~3-fold) (Figures 2G and S4D–S4F). Robo3 is a transmembrane protein that, during development, is localized to axons and is required for proper patterning of the nervous system.^63^ Thus, T3 induction of *Robo3* in neurons and of many developmental- and synapse-associated genes across cell types suggest that thyroid hormone drives a concerted program to induce plasticity of cortical circuits.

### T3 acts within glutamatergic projection neurons to alter their transcriptome

**Figure 3.**
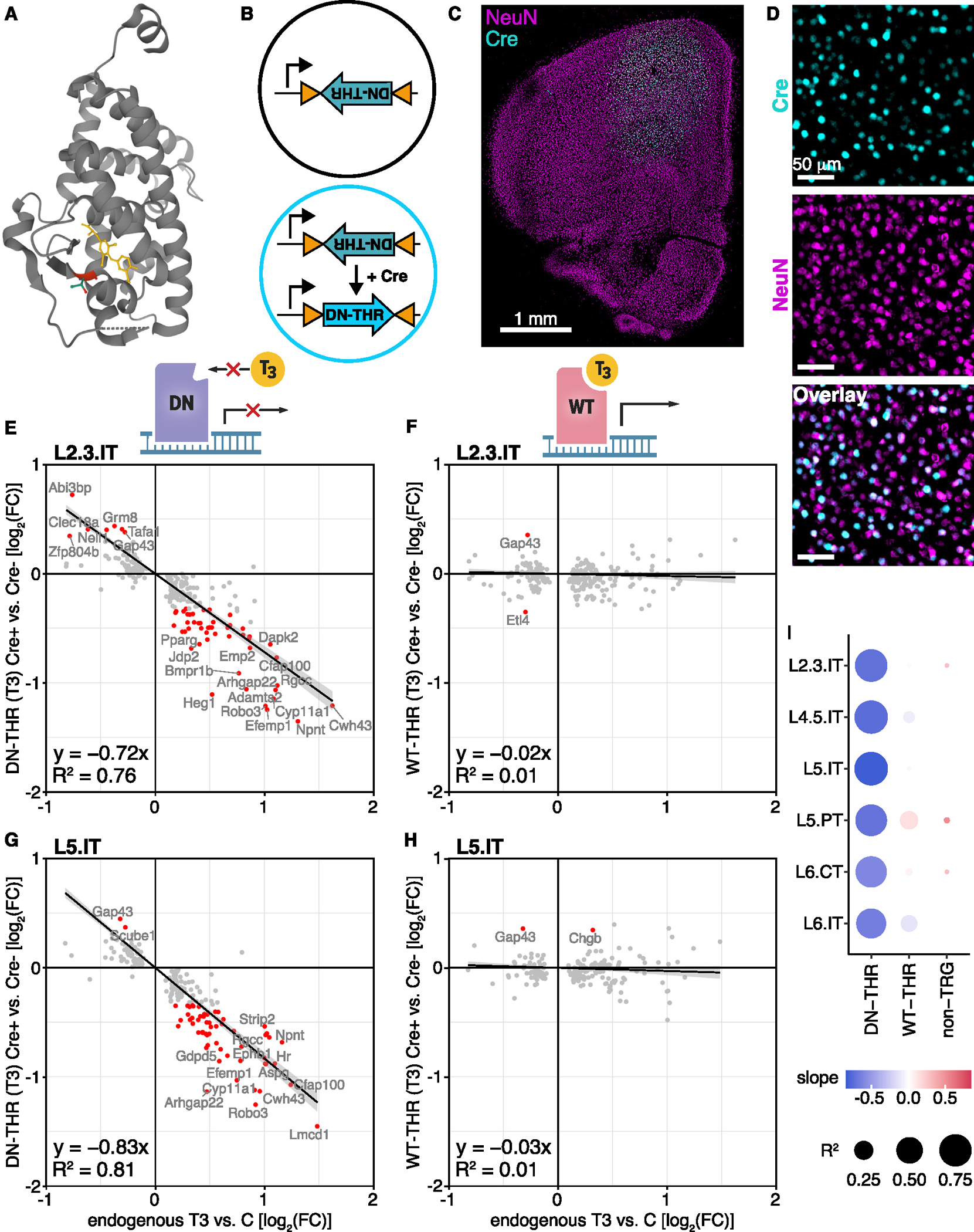
T3-induced transcriptional programs are due to direct T3 action on its receptors (A) Crystal structure of human THR bound to T3 (PDB: 3GWS). Threonine 337 is highlighted in red; its deletion prevents T3 binding. (B) Schematic of viral strategy. An AAV driving expression of a Cre-dependent DN-THR was transduced broadly. A second self-complementary AAV driving expression of Cre was delivered at low infectious titer to produce mosaic tissue in which only a subset of neurons express Cre and activate expression of DN-THR. (C) Image of cortical hemisphere showing a representative injection with expression of Cre (cyan) labeled by an HA tag, centered on M2, and all neurons labeled by neuronal peri-nuclei (NeuN) (magenta). Scale bar, 1 mm. (D) Magnified images within the injection site, showing Cre-expressing neurons (top: cyan, HA labeled) and all neurons (middle: magenta, NeuN labeled), and their overlay (bottom). We found Cre expressed in 44% of neurons (1,639/3,708 NeuN-labeled cells were also HA positive, from *n* = 2 animals). Scale bars, 50 μm. (E) Plot showing on the y axis the log_2_(fold-change) of L2.3.IT TRGs between Cre+ (DN-THR expressing) L2.3.IT cells and Cre− (lacking DN-THR) L2.3.IT cells after T3 treatment. The x axis shows the log_2_(fold-change) of L2.3.IT TRGs between the T3 and vehicle control conditions from the original dataset (Figure 2). Red dots highlight TRGs whose expression was significantly disrupted due to DN-THR (FDR-adjusted *p* < 0.05, and fractional change in expression of at least ±25%). Lines/shade: linear regression fit/95% confidence interval. Fit equation and R^2^ value are displayed. See Figure S4G. (F) As in (E), but for WT-THR expressing tissue. See Figure S4H. (G) As in (E), but for L5.IT neurons and L5.IT TRGs. (H) As in (F), but for L5.IT neurons and L5.IT TRGs. (I) Dot plot of linear regression fits across glutamatergic projection neurons. DN-THR expression disrupted TRG programs, resulting in a large negative slope across cell types. This indicated that normally upregulated TRGs are downregulated by DN-THR, and normally downregulated TRGs are upregulated by DN-THR. By contrast, slopes were near zero and had low R^2^ for comparisons between WT-THR expressing and lacking cells, indicating that over-expression of the functional receptor does not broadly disrupt TRG programs. Similarly, DN-THR did not disrupt non-TRG expression. See also Figure S4.

The changes in neuronal gene expression in M2 could result from thyroid hormone action elsewhere in the brain. For example, T3 effects in the hypothalamus could result in secretion of a factor that subsequently alters cortical transcription. Alternatively, neurons may respond indirectly to T3-activated programs within neighboring non-neuronal cells. To address whether the T3-regulated transcriptional programs are a cell-autonomous response to T3 in cortical neurons, we utilized a thyroid receptor β (THRβ) mutant derived from an individual with generalized thyroid resistance.^64^ The DNA binding domain is intact, but the receptor has a single amino acid deletion (threonine 337) that prevents it from binding thyroid hormone and activating transcription^65^ (Figure 3A). Therefore, the mutant THR acts as a dominant-negative (DN), competing for DNA binding sites, which are largely shared between THRα and THRβ,^66–68^ and blocking transcriptional activation in response to T3. Unliganded THRs bind to DNA and recruit transcriptional regulators, an aspect of THR biology that is preserved in the DN-THR but lost with genetic deletion of THRs.^68^

We injected two adeno-associated viral vectors (AAVs) in M2. One drove expression of a Cre-dependent DN-THR. The other encoded Cre driven by the neuron-active human synapsin promoter and was delivered at low titer for stochastic transduction.^69^ This strategy created mosaic tissue with a subset of neurons expressing DN-THR. After 2 weeks for receptor expression, we treated animals with T3, dissected the tissue, and performed snRNA-seq. Cre transcripts were detected in ~44% of neurons, similar to the proportion of Cre positive neurons detected by immunohistochemistry (Figures 3B–3D).

We compared the expression of TRGs with and without DN-THR (defined by the detection of Cre) in glutamatergic projection neurons, which as a neuronal class had the largest sample size and induced the most TRGs. DN-THR significantly dampened the transcriptional response to T3 across all glutamatergic projection neuron classes (Figures 3E–3G and S4G). For instance, in layer 2/3 (L2/3) and layer 5 (L5) intratelencephalic (IT) neurons, linear regressions comparing changes in TRGs due to DN-THR vs. endogenous TRG induction resulted in large negative slopes showing that DN-THR downregulates the expression of normally upregulated TRGs and upregulates the expression of normally downregulated TRGs. Non-T3-regulated genes were minimally affected (R^2^ ≤ 0.02 across cell types) by DN-THR, which, overall, significantly modulated levels of only 1% of genes by 25% or more (Figures 3I and S4I).

To confirm that disrupted TRG expression was due to the inability of DN-THR to bind T3 and activate transcription, we repeated the experiments with wild-type (WT)-THR. In contrast to DN-THR, expression of the WT-THR maintained the TRG program, reflected by linear regression slopes near zero and a lack of significantly disrupted TRGs (Figures 3F–3I and S4H). The non-perturbative nature of WT-THR over-expression suggests that, in cortical neurons, thyroid hormone signaling is limited by levels of T3, not THRs.

Thus, DN-THR expression in individual glutamatergic projection neurons is sufficient to perturb their TRG transcriptional programs, indicating these transcriptional changes result from T3 activating receptors within each neuron, as opposed to indirectly due to T3 effects on other brain regions or cell types. Furthermore, they confirm that these genetically encoded tools can be used to perturb T3-regulated gene expression in specific cell types and brain regions.

### T3 induces cell-type-specific plasticity of synaptic transmission in cortex

**Figure 4.**
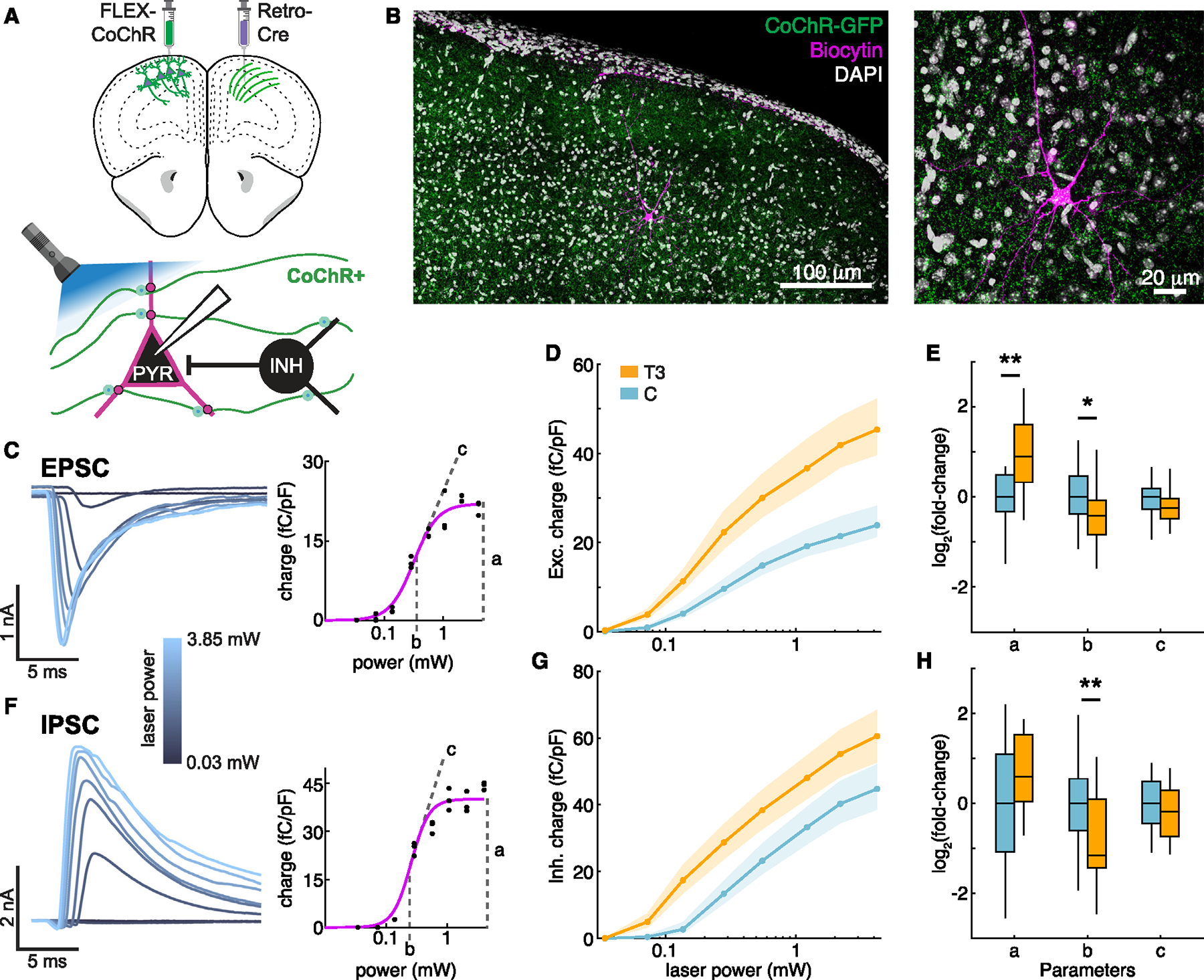
T3 alters synaptic connectivity of cortical glutamatergic neurons (A) Top: AAV encoding Cre-dependent CoChR (FLEX-CoChR) was delivered to the upper layers of M2 in one hemisphere, and a retrograde AAV encoding Cre was delivered to the contralateral hemisphere, resulting in CoChR expression in neurons that send projections to contralateral M2. Bottom: whole-cell recordings from contralateral L2/3 pyramidal neurons within the field of CoChR-expressing axons were used to measure PSCs triggered by whole-field optogenetic stimulation. (B) Low-magnification (left; scale bar, 100 μm) and enlarged (right; scale bar, 20 μm) images of a streptavidin-labeled L2/3 pyramidal neuron filled with biocytin (pink) via the recording pipette amidst CoChR-expressing axons (green). (C) Left: example of light-evoked EPSCs. Currents are color coded by the light stimulus intensity. Right: excitatory charge in a post-stimulus 10 ms window normalized by cell capacitance as a function of light stimulus power. Data were fit by a sigmoid (pink) characterized by a saturation amplitude (a), a half-maximum inflection point (b, measure of sensitivity), and the slope (c). (D) Normalized post-synaptic excitatory charge as a function of laser stimulus power for T3 (orange, *n* = 21 neurons, 8 mice) and vehicle (blue, *n* = 29 neurons, 10 mice) treated mice. Dots/shade: mean/bootstrapped SEM. (E) Boxplot of changes in sigmoid parameter (from single-cell fits of excitatory charge vs. laser power curves) relative to the median control value. T3-treatment significantly increased the saturation amplitude (a, *p* = 0.001) and decreased the power to half-maximum (b, *p* = 0.04), without changing the slope (c, p = 0.10). Central line: median, box: IQ, whiskers: data within 1.5× IQR. (F–H) As in (C)–(E), but for light-evoked IPSCs and normalized inhibitory charge. T3 treatment significantly decreased the power to half-maximum IPSC charge (b, *p* = 0.005) but did not increase the saturation amplitude (a, *p* = 0.09) or change the slope (c, *p* = 0.35). All statistical comparisons: Wilcoxon rank-sum test. *p < 0.05, **p < 0.01. See also Figure S5.

Given the T3-dependent induction of axon- and synapse-related genes, we examined if T3 affects cortical circuits and synapses. Upper layer IT glutamatergic neurons that project to contralateral M2 are critical to decision-making as well as motor planning and execution.^70,71^ We expressed a channelrhodopsin variant, CoChR-GFP,^72^ in these neurons in one hemisphere of M2 through retrograde labeling of their anatomical projections (Figure 4A). We then performed whole-cell recordings of L2/3 pyramidal neurons in contralateral M2 within the CoChR-GFP labeled axonal field in acute brain slices prepared from T3-treated and control animals (Figures 4A–4C; Table S3). We varied the intensity of blue-light stimulation over two orders of magnitude to characterize post-synaptic currents (PSCs) across the full range of optogenetic stimulation. PSCs were measured at the reversal potentials of glutamate- and GABA-gated ion channels to isolate inhibitory and excitatory currents. The resulting data from each neuron were fit to a sigmoid to obtain a response-profile relating light power to the magnitude of evoked currents.

T3 treatment increased excitatory PSCs (EPSCs) across the full range of light intensities: parameterizing these responses revealed that T3-treatment significantly increased the amplitude of saturating post-synaptic responses and sensitized the current to light power, reducing the light required for half-maximum stimulation (Figures 4D and 4E). By contrast, di-synaptic inhibitory PSCs (IPSCs) onto L2/3 neurons had a significant increase in sensitization but no increase in saturating amplitude in response to T3 (Figures 4F–4H). The changes in EPSCs were unlikely to be due to alterations in intrinsic excitability of presynaptic neurons, as T3 treatment affected neither passive nor active properties of L2/3 pyramidal neurons (Figures S5A and S5B; Table S3). Separate experiments, using light intensities titrated to produce similarly sized EPSCs in each L2/3 pyramidal neuron showed that optical paired pulse ratios (PPRs) were unaffected by T3, suggesting similar vesicle release probability from trans-hemispheric axons of IT neurons across conditions (Figure S5C).

We examined if the sensitization of di-synaptic IPSCs was due to direct effects of T3 on GABAergic interneurons recruited by trans-hemispheric glutamatergic axons. We expressed channelrhodopsin in parvalbumin (PV) interneurons within M2,^73^ which are rapidly recruited by cortical excitation^74^ and had the most TRGs among GABAergic neuronal cell types. We repeated whole-cell recordings of L2/3 pyramidal neurons while stimulating PV neurons. T3 treatment did not alter optically elicited IPSCs (Figures S5D–S5F), suggesting that the sensitization of di-synaptic IPSCs on L2/3 pyramidal neurons by T3 is due to altered synaptic input onto PV neurons, as opposed to changes in neurotransmission by PV neurons. Thus, increasing T3 levels alters synapses in a cell-type-specific manner to remodel cortical circuits.

We examined if T3-dependent transcriptional programs were required in the presynaptic cell to modulate synaptic connectivity. We co-expressed CoChR-GFP along with WT-THR or DN-THR in the neurons that send projections to contralateral M2 to specifically disrupt T3-dependent transcriptional cascades within the presynaptic neurons and then repeated the electrophysiology analysis of PSCs in recipient L2/3 pyramidal neurons. The previously observed effects of T3 were recapitulated in experiments with presynaptic expression of WT-THR (Figures S5G–S5I), consistent with the lack of prominent effects of WT-THR expression on T3-dependent transcription. However, presynaptic expression of DN-THR occluded the effects of T3 (Figures S5J–S5L), indicating that intact T3-dependent transcriptional programs in the presynaptic neurons are required for T3-induced changes in synaptic transmission.

Finally, we performed similar experiments in *Robo3*^*fl/fl*^ mice, introducing Cre or an inactive Cre control (ΔCre) into one hemisphere along with CoChR-GFP, and then repeated the electrophysiology analysis of PSCs in contralateral L2/3 pyramidal neurons. ΔCre expression affected neither *Robo3* expression nor PSC changes induced by T3 (Figures S5M–S5O). By contrast, loss of *Robo3* occluded the effects of T3 on EPSCs and di-synaptic IPSCs (Figures S5P–S5R), indicating that changes in *Robo3* expression in presynaptic glutamatergic neurons plays a critical role in T3-induced plasticity of cortical circuits.

### T3 alters decision-making in a probabilistic simulated foraging task

We examined if thyroid-dependent changes in cortical transcription and circuitry directly impact exploratory behaviors. Mice expressing DN-THR in neurons of frontal cortices did not significantly increase time spent in the exposed region of the LD assay with T3 treatment (Figures S6A–S6F), suggesting that T3-dependent transcriptional regulation in cortical neurons alters exploratory-like behaviors. However, it is unclear what cognitive processes underlying such behaviors are influenced by T3-dependent transcriptional programs.

**Figure 5.**
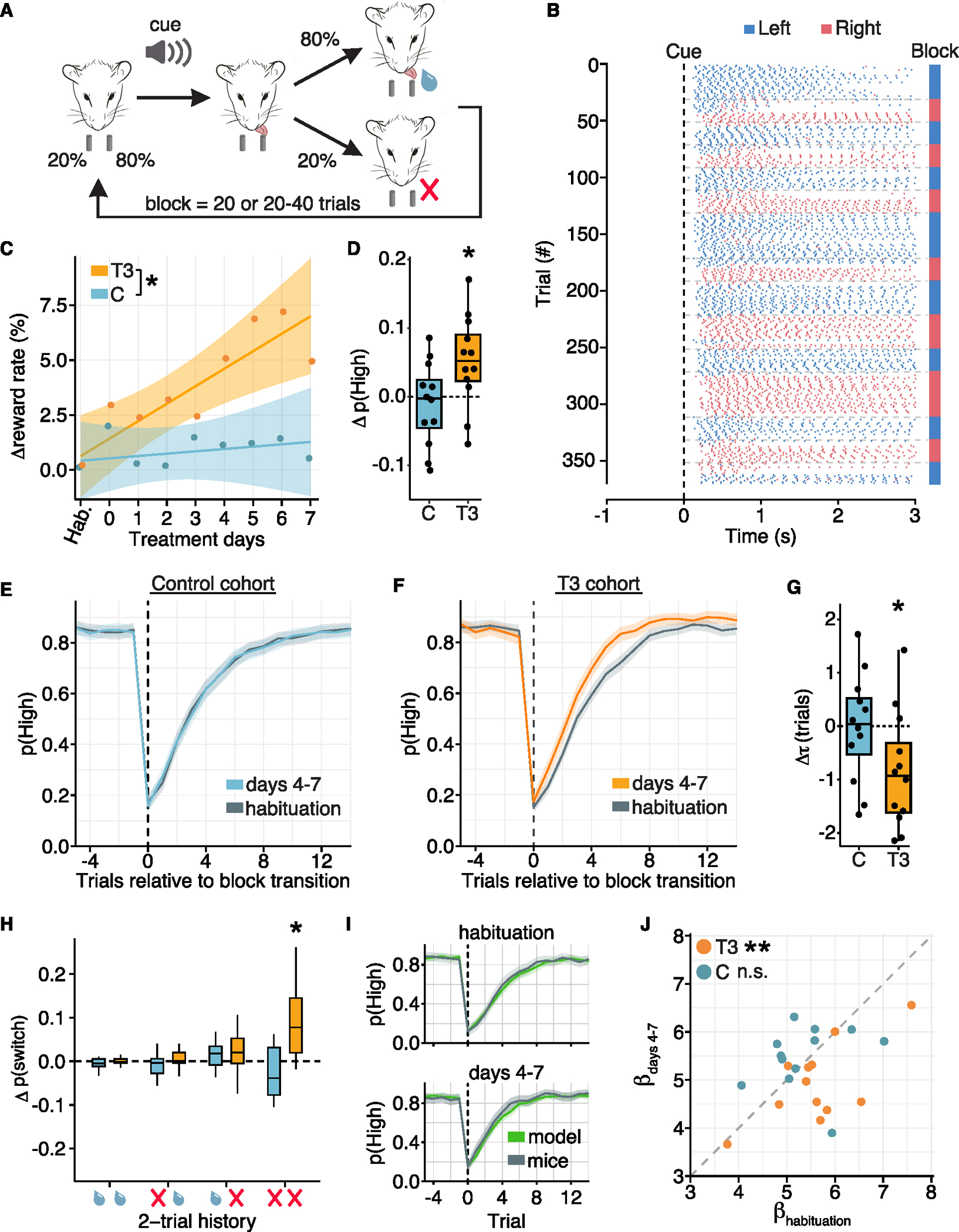
T3 alters decision-making and exploration in the 2-armed bandit task (A) Schematic of the 2ABT. On each trial, one spout is likely to dispense a water droplet (80%), and the other spout is unlikely (20%). A tone (5 kHz) cues the start of the selection period, during which a mouse can make a choice by licking one of the two spouts. The mouse then receives water drops according to its spout choice and reward probabilities. Reward probabilities are dynamic, switching without cue after a block of 20 trials for data presented in (C)–(J), or blocks of 20–40 trials for that in Figures 6C–6I. (B) Raster plot from a 2ABT session (blocks of 20–40 trials) showing individual licks to left (blue) and right (red) spouts as a function of time from start of tone, marking the selection period (black dotted line). Color code (right) indicates the identity of the highly rewarding spout. Gray dotted lines mark block transitions. (C) Percent change in reward rate (rewards/trial) relative to habituation days (Hab.) for T3 (orange) and vehicle (blue) treated mice. The reward rate of each mouse was normalized to the median rate during habituation. Dots indicate the average change in reward rate across mice per day. Lines/shade: linear fits/95% confidence intervals. There was a significant interaction of treatment condition and change in reward rate over the experiment (*p* = 0.03, likelihood ratio test), and the rate increased with T3 treatment (linear regression, F = 12.42 (1,134), *p* < 10^−3^), but was stable with control treatment (linear regression, F < 10^−3^ (1,139), *p* = 0.62). Normalized reward rate of T3-treated animals significantly increased on and after 4 days of treatment (day 0: *p* = 0.21, 1: *p* = 0.34, 2: *p* = 0.17, 3: *p* = 0.32, 4: *p* = 0.02, 5: *p* = 0.007, 6: *p* = 0.001, 7: *p* = 0.04; likelihood ratio test). (D) Change in probability of selecting the highly rewarding spout, p(High), between the habituation period and treatment days 4–7 calculated as the differences of median values. Black dots: single mice. T3-treated mice significantly increased p(High) (*p* = 0.02), whereas vehicle-treated mice did not (*p* = 0.55). Paired t tests. (E) p(High) as a function of trial position within a block for vehicle-treated mice during habituation (gray) or treatment days 4–7 (blue). Trial 0 marks the first trial of a new block. Shading: 95% confidence intervals. (F) As in (E) but for T3-treated mice (treatment days 4–7, orange). (G) Change in the time constant (τ) from exponential fits to p(High) after the block transition between habituation and treatment days 4–7. Black dots: single mice. T3-treated mice had a significant decline in τ (*p* = 0.02); vehicle-treated mice did not (*p* = 0.93). Paired t tests. (H) Change in conditional switch probabilities, dependent on reward outcomes of the previous 2 trials, between habituation and treatment days 4–7. The 4 most common histories are plotted, which resulted from selecting the same spout on two consecutive trials with varying reward outcomes, represented by a water droplet (reward) or red X (no reward). T3-treated mice increased their probability of switching spouts in response to two consecutive failures (*p*-adjusted = 0.02). No other conditional switch probabilities changed (*p*-adjusted > 0.05). Paired t tests with Benjamini-Hochberg correction. (I) Q-learning model predictions on held-out data of p(High) around block transitions from habituation (top) and days 4–7 (bottom). Gray line is mean probability from the mouse data (T3 cohort); green line is the model prediction. Shading: 95% confidence intervals. The model fit the data well for all treatments and epochs (for T3 cohort, spout-choice prediction accuracy on held-out data during habituation: 0.85 ± 0.03, mean ± SD; days 4–7: 0.85 ± 0.03; comparison between epochs: *p* = 0.52; for control cohort, spout-choice prediction accuracy on held-out data during habituation: 0.85 ± 0.03; days 4–7: 0.86 ± 0.03; comparison between epochs: *p* = 0.64; paired t tests). (J) Scatterplot of β parameter fits during habituation (x axis) and days 4–7 of treatment (y axis) for each animal. T3-treated mice had a significant decrease in β between habituation and days 4–7 (*p* = 0.008), whereas vehicle-treated mice did not (*p* = 0.69). Paired t test. For all analyses, *n* = 12 animals for each treatment condition (T3 or control). *p < 0.05, **p < 0.01. For all boxplots, central line: median, box: IQ, whiskers: data within 1.5× IQR. See also Figure S6.

We hypothesized that T3-dependent transcriptional programs and subsequent changes in cortical circuitry influence flexible decision-making and evidence accumulation, key cognitive processes underlying exploration that are typically cortex dependent.^75^ To test this, we utilized the 2-armed bandit task (2ABT), which simulates animal foraging, a prototypical exploratory behavior^76^ that requires the animal to integrate and weigh information to make decisions in an uncertain and dynamic environment and, in mice, requires anterior regions of cortex.^77–81^ Thirsty, head-restrained mice were required to choose between two spouts located on each side of their heads that deliver water with probabilities that change over time (Figure 5A). On each trial, a tone marks the beginning of the selection period, during which a mouse can make a choice by licking one of the two spouts. On each trial, one spout, if selected, delivers a drop of water with high probability (0.8), while the other delivers water with low probability (0.2). However, the reward contingencies of the spouts reverse—without any sensory cue—after a block of trials. Mice learned to perform this task and explored both spouts to accumulate evidence about the reward status of the environment (Figure 5B).

To determine whether T3 influences exploratory decision-making, we trained animals to proficiency, then treated animals with vehicle solution for multiple sessions (habituation period) to establish a baseline, and finally treated animals with T3 or continued vehicle administration (control). Motor action measures, such as spontaneous lick rate and reaction times, were not altered by T3 (Figures S6G–S6I). However, mouse performance, as measured by the reward rate, improved with T3. The reward rate increased with T3 over the 8 days of treatment and was significantly increased by day 4 (Figure 5C), consistent with a transcriptional rather than an acute mechanism. By contrast, performance was stable in the control cohort (Figure 5C). The T3-induced increase in reward rate was driven by an increase in the probability of choosing the highly rewarding spout, p(high), which was apparent when comparing performance during the habituation period and treatment days 4–7 (Figure 5D). To understand what changes in behavior underlaid improved task performance, we examined p(high) as a function of trial position within a block: mice with elevated T3 switched more rapidly to predominantly selecting the new highly rewarding spout after block transitions, reflected by a ~30% smaller time constant from exponential fits of the data (Figures 5E–5G).

The switch to the new highly rewarding spout in fewer trials after the block transition suggests that animals alter how they integrate information across trials to infer the highly rewarded spout. Therefore, we examined the conditional probability of switching spouts after the 4 most highly occurring 2-trial histories (>50/mouse) during habituation and treatment days 4–7. Each 2-trial history resulted from licking the same spout in consecutive trials with varying reward outcomes. T3-treated mice significantly increased the spout switch rate in response to two consecutive failures to receive a water droplet, but not in response to the other 2-trial histories (Figure 5H). Thus, T3 promotes spout switching dependent on previous choice outcomes that imply a change in reward contingencies.

This paradigm requires mice to accumulate evidence to make inferences about the environment and use that inference along with a policy to choose a spout. We modeled this history-dependent decision-making using a Q-learning reinforcement framework, which separates the inference and policy processes^78,82,83^ and whose variables are represented in the firing activity of anterior cortical neurons.^78^ The model fit the data well, both prior to and during T3 treatment (Figure 5I). The model parameters include learning (α) and forgetting (ζ) rates for chosen and unchosen spouts, neither of which changed with T3 treatment (Figures S6J–S6L), suggesting that T3 did not alter rates of evidence accumulation or storage. The only parameter that changed with T3 was β, which characterizes the degree to which decisions exploit current evidence relating to which spout is highly rewarding vs. exploring the spouts stochastically to gain new information. β significantly decreased with T3, enabling animals to switch spouts more rapidly at block transitions and improve their overall task performance (Figure 5J). Thus, mice with increased T3 change their decision-making policy to explore the alternate spout with less evidence that the environment has changed.

**Figure 6.**
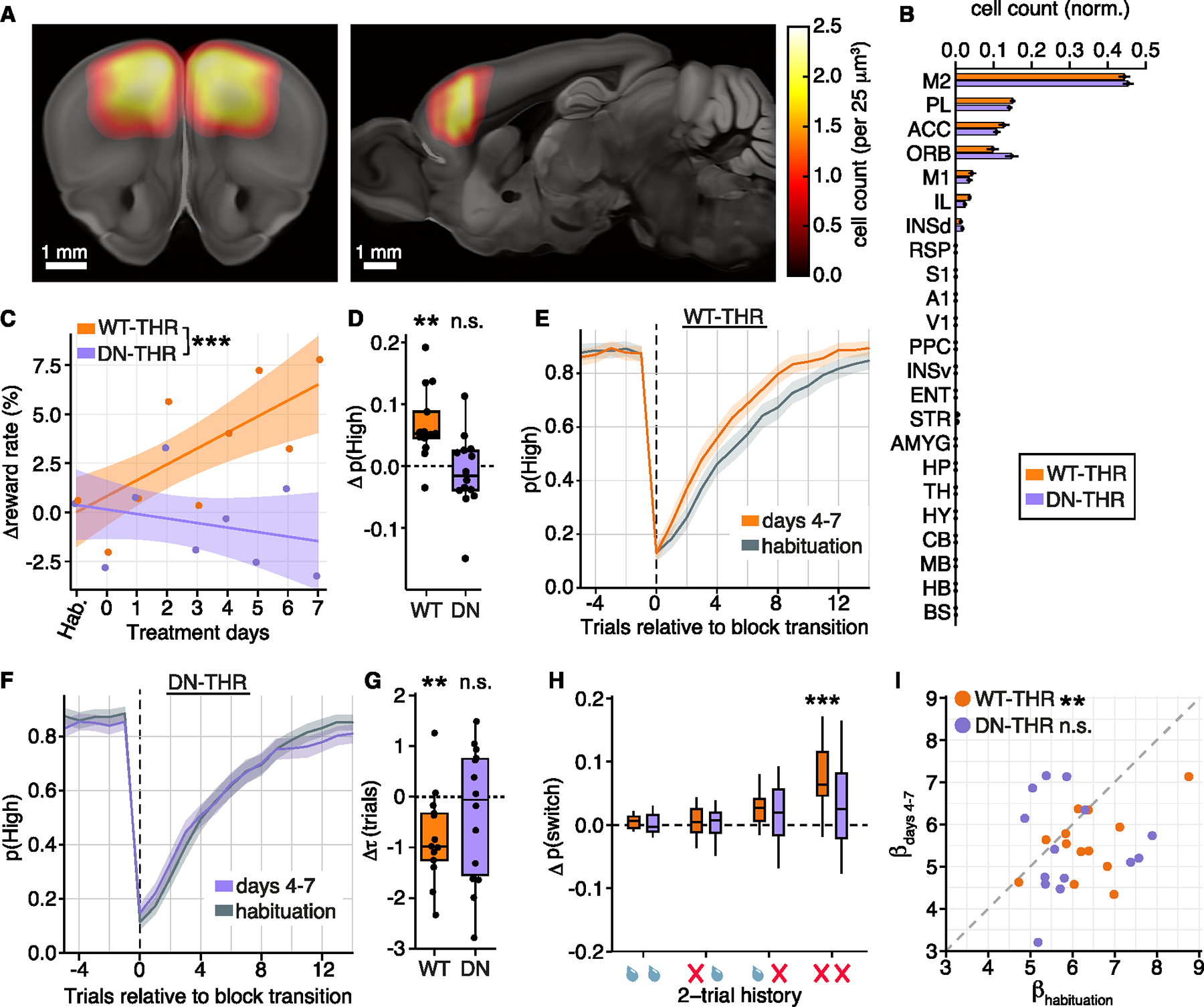
T3-dependent transcriptional cascades in frontal cortex neurons underlie T3 mediated changes in exploratory decision-making (A) Average heatmaps of DN-THR expression in the cohort of mice performing the 2ABT. Scale bars, 1 mm. Left: coronal section ~2 mm anterior to bregma. Right: sagittal section ~1 mm from midline. An extended selection of heatmaps of both DN-THR and WT-THR animals is in Figure S6M. (B) Bar plot of the normalized count of WT-THR (orange) and DN-THR (purple) cells across brain regions. Brain region expression was similar between WT-THR and DN-THR cohorts (Table S4). Bar/error bar: mean/SEM. (C) The percent change in reward rate (rewards/trial) over habituation and treatment. All habituation days are grouped (Hab.), and the reward rate of each mouse was normalized to the median rate during habituation. Dots indicate the average change in reward rate per day for WT-THR (orange) and DN-THR (purple) animals. Both cohorts received T3. Lines/shade: linear fits/95% confidence intervals. There was a significant interaction of genotype (WT-THR/DN-THR) and treatment duration (*p* < 10^−3^, likelihood ratio test). WT-THR animals increased their reward rate with T3 treatment (linear regression, F = 14.14 (1,154), *p* < 10^−3^), while DN-THR animals did not (linear regression, F = 1.11 (1,166), *p* = 0.29). (D) Change in p(High) between the habituation period and treatment days 4–7 calculated as the difference of median values from each period. WT-THR mice significantly increased p(High) (*p* = 0.001), DN-THR mice did not (*p* = 0.54). Paired t tests. (E) p(High) for WT-THR animals as a function of trial position within a block. Orange line: mean probabilities over days 4–7. Gray line: mean probabilities over habituation days. Shading: 95% confidence intervals. (F) As in (E) but for DN-THR animals (treatment days 4–7, purple). (G) Change in time constant (τ) of recovery of p(High) from exponential fits of the data (aligned to the block transition) between the habituation period and days 4–7. WT-THR mice had a significant decline in τ (*p* = 0.009), DN-THR mice did not (*p* = 0.35). Paired t tests. (H) Change in conditional switch probabilities between the habituation period and days 4–7 (as in Figure 5H). WT-THR mice increased their probability of switching spouts in response to two consecutive failures (*p*-adjusted < 10^−3^); DN-THR did not (*p*-adjusted = 0.40). No other conditional switch probabilities changed (*p*-adjusted > 0.05). Paired t tests with Benjamini-Hochberg correction. (I) Scatterplot of β parameter fits during habituation (x axis) and treatment days 4–7 (y axis) for each animal. WT-THR mice significantly decrease β between habituation and days 4–7 (*p* = 0.007); DN-THR mice did not (*p* = 0.28). Paired t tests. For post hoc histology and quantification in (A) and (B), WT-THR cohort: *n* = 12 animals, DN-THR cohort: *n* = 10 animals. For all other analyses, WT-THR cohort: *n* = 13 animals, DN-THR cohort: *n* = 14 animals. **p < 0.01, ***p < 0.001. Black dots represent data from single mice. For all boxplots, central line: median, box: IQ, whiskers: data within 1.5× IQR. See also Figure S6.

Finally, we examined whether the changes in behavior depended on the neuron-specific TRG program in anterior cortical regions. We introduced AAVs to express DN-THR or WT-THR in neurons centered on M2 in frontal cortex (Figures 6A, 6B, S6A–S6E, and S6M; Table S4). Expression of DN-THR did not perturb T3-sensitive aspects of physiology that may be relevant to the 2ABT, including thirst, a motivating drive during the 2ABT, and heart rate, which can influence behavioral state^84^ (Figures S6N–S6Q). Therefore, we trained a new cohort of animals to proficiency in 2ABT, then introduced DN-THR or WT-THR to frontal cortex and continued training the animals for ~2 weeks to allow for THR expression. We carried out the same paradigm as before—habituation followed by 8 days of T3 treatment. As above, T3 did not alter gross motor actions such as spontaneous lick rate or reaction times in either WT-THR or DN-THR cohorts (Figures S6R–S6T). The WT-THR cohort recapitulated the previously observed effects of T3 on performance: an increasing reward rate over treatment sessions, mediated by an increased probability of selecting the highly rewarding spout (Figures 6C and 6D). Animals more rapidly switched their selections after block transitions with T3 and altered their probability of switching dependent on previous trial outcomes (T3 treatment significantly increased switching after two consecutive failures) (Figures 6E–6H). Finally, in the Q-learning model, β declined with T3, while all other parameters remained unchanged (Figures 6I and S6U–S6Y).

By contrast, performance of DN-THR expressing animals did not change in response to elevated T3. The DN-THR cohort had no increase in reward rate over the experimental time course, did not change their probability of selecting the highly rewarding spout, and did not alter their trial-outcome dependent switching probabilities (Figures 6C–6H). In addition, there were no significant changes in any Q-learning model parameters, including β after T3 administration (Figures 6I and S6U–S6Y). Thus, although THRs are expressed in many brain areas that likely act in concert to coregulate T3-dependent changes in exploratory behaviors, T3-dependent transcriptional cascades in neurons within frontal cortex are necessary for thyroid-mediated changes in exploratory decision-making. These studies reveal a mechanism by which a hormonal sentinel of environmental change directly affects neuronal circuits in cortex to drive adaptive changes in exploratory drive.

## DISCUSSION

Variation in thyroid hormone levels can have profound effects on human behavior ranging from depressed-like states with pathologically reduced thyroid function to manic or even psychotic states with pathologically elevated thyroid hormone in individuals with hypo- and hyper-thyroidism, respectively.^30–33^ Similar behavioral changes occur in wild and domesticated animals with physiological seasonal variation in thyroid function, leading to adaptive changes in exploratory behavior in synchrony with environmental variables such as food availability and weather. Here, we examine the mechanisms of such changes and reveal a T3-sensitive transcriptional program induced in cortical neurons of male mice that alters cortical circuits and increases exploration and risk taking in a variety of contexts. We find that cortical axon-pathfinding transcriptional programs are reawakened and dynamically modulated in adult cortex by thyroid hormone and serve a physiological function to link peripheral metabolism to central exploratory drive. Pharmacological manipulation of such programs may have therapeutic value for the treatment of neuropsychiatric disorders such as depression and bipolar disorder (BD) in which exploratory drive is dysregulated.

### T3-induced plasticity of adult cortical circuits via cell-type-specific gene regulation

The transcriptional signatures that demarcate the dozens of molecularly defined cell types within cerebral cortex are not static—they change over development and aging,^85,86^ and respond dynamically to life experiences.^87,88^ Nuclear hormones contribute to these dynamic signatures, as they fluctuate in response to experience, environment, age, and internal somatic states—over timescales ranging from minutes to seasons to lifespans^89–91^—to impact cortical cell types and circuits.^5,92–94^

We find that thyroid hormone, known to regulate the metabolic state of peripheral organs, induces cell-type-specific transcriptional programs in adult cerebral cortex in a manner that, at least in glutamatergic projection neurons, is largely cell-autonomous and driven by local T3 levels. These programs are tailored to the function of each cell type. For instance, glutamatergic projection neurons engage programs highly enriched for molecules involved in axonal remodeling, whereas transcriptional programs in both astrocytes and neurons are enriched for molecules involved in assembling and regulating synapses. In oligodendrocytes, T3 induced pathways related to their differentiation, maturation, and myelination.

The neuronal T3-induced gene regulatory programs alter excitation and recruitment of polysynaptic inhibition in L2/3 pyramidal neurons evoked by trans-hemispheric projections, indicating remodeling of adult cortical circuits. T3-induced transcriptional changes, including induction of the axon-pathfinding gene *Robo3*, are necessary specifically in presynaptic neurons for thyroid-dependent synaptic changes. T3 alters neither paired-pulse ratios of excitation nor direct PV interneuron-evoked GABAergic currents in L2/3 pyramidal neurons, suggesting a primary effect on the potency or number of glutamatergic synapses. Consistent with the function of the induced genes, the substrate for this plasticity could include new synapse formation on existing axons or potentially new axon branches.

Given the induction of pro-myelination genes by T3 in oligodendrocytes and the activation of synaptic regulators in astrocytes, non-neuronal cells likely contribute to circuit remodeling. Further, the absence of observed T3-induced plasticity of PV interneuron to L2/3 pyramidal neuron GABAergic transmission suggests that cell-type-specific T3-dependent transcriptional programs translate to cell-type-specific changes in synaptic transmission. A full understanding of the function of T3 target genes in each cell type will reveal the molecular and anatomical mechanisms driving novel forms of hormonally induced adult plasticity in cerebral cortex.

### T3 coordinates exploration and metabolism

T3 induced changes in the exploratory behaviors of male mice in several paradigms, including in spontaneous exploration of behavioral syllable sequences expressed in a featureless arena, in coarse examinations of exploration in a LD preference assay, and within the theoretically motivated and learned 2ABT, designed to probe dynamic exploratory decision-making with evidence accumulation. The 2ABT allows for the measurement and modeling of how an animal integrates and weighs information in an uncertain environment—key cognitive processes that underlie exploratory decision-making. Doing so revealed a specific T3-induced increase in exploratory decision-making that is reliant on T3-driven transcriptional programs in neurons of anterior cortex. Coupled with our findings that these programs contribute to innate exploration in the LD assay, we conclude that these neuronal T3-driven transcriptional programs causally drive changes in exploratory drive, such that higher levels of T3 synchronously increase body-wide metabolism and expression of exploratory, information-seeking behaviors.

The coordinated response of body and brain to thyroid hormone is likely adaptive. We propose that in environments with seasonal changes in food availability, an increase in thyroid hormone signals an increase in the expected abundance of resources. The sensory cues promoting changes to thyroid hormone signaling remain to be fully elucidated and may vary between species, but drivers include photoperiod, temperature, and diet.^21,95–97^ Regardless of sensory triggers, during the time of year when food is available, animals’ thyroid hormone levels rise, increasing their energy expenditure and willingness to explore and forage. Conversely, when resources are scarce, thyroid hormone levels drop, slowing metabolism and biasing animals toward energy conserving behaviors. Indeed, thyroid hormone levels are known to drop in response to food restriction and deprivation,^98^ and low T3 levels within hypothalamus are likely critical for maintaining a state of hibernation.^16,99^ Thus, the local actions of T3 in different organs across the body coordinate metabolic state with adaptive changes to exploratory drive.

### Cortical circuits that contribute to mood disorders

Our findings suggest that changes in T3 levels are a natural perturbation that modulates cortical circuitry along physiologically relevant axes, resulting in altered exploratory drive. Such evolutionarily conserved programs,^100^ including thyroid hormone-dependent control of behavior, can be exploited to reveal canonical brain circuit functions that go awry in psychiatric disorders in which exploratory drive is dysregulated. These include symptoms of depression featuring pathological neglect of exploratory behaviors and symptoms of mania and risk taking present in BD. Intriguingly, T3 has been used as an effective therapy for depression in humans^101^ independent of thyroid status, supporting our results that thyroid hormone levels regulate exploratory drive. Additionally, symptomatic profiles of BD and hyperthyroidism overlap. Individuals with BD have increased prevalence of thyroid disease,^102^ treatment with lithium is known to significantly perturb thyroid organ function^103^ and thyroid receptor expression in the brain,^104^ and thyroid modulators can be effective for BD management.^105^ More broadly, there is a high incidence of metabolic disorders, such as diabetes mellitus, in individuals with BD^106,107^ and depression,^108–110^ suggesting a perturbed link between exploratory behavior and metabolism. Despite etiological differences, the challenges of modeling mood disorders using monogenic animal models motivate a search for convergent mechanisms that could exist at the molecular, cellular, and circuit levels between these disorders, providing a path to search for conserved targets for disease treatment.

### Limitations of the study

Female mice did not show a statistically significant change in behavior in the LD assay with T3-administration, suggesting important sex-dependent differences in thyroid hormone signaling and potential downstream changes to physiology and behavior, as observed across many species.^17,20–29,41^ Differences in thyroid hormone signaling could arise from sex-dependent regulation of thyroid hormone bioavailability or from direct changes in the transcriptional or physiological response to T3 in the brain. Precisely defining T3-driven behavioral variation in females will be vital given the increased prevalence, secondary to autoimmune disorders, of thyroid illness in females and the overall lack of understanding of the effects of thyroid hormone on human behavior.

### Conclusions

Our studies reveal how the action of a single hormone can coordinate two seemingly disparate biological phenomena: exploratory drive and whole-body metabolic state. We anticipate that the systematic characterization of the vast array of hormonally driven molecular programs in cerebral cortex will reveal a range of novel circuit plasticity mechanisms to tune complex behaviors to match the needs of the body and the demands of the environment.

## STAR★METHODS

### RESOURCE AVAILABILITY

#### Lead contact

Further information and requests for resources and reagents should be directed to and will be fulfilled by the lead contact, Bernardo Sabatini (Bernardo_sabatini@hms.harvard.edu).

#### Materials availability

Plasmids generated in this study will be available at the time of publication on Addgene (https://www.addgene.org/Bernardo_Sabatini/).

#### Data and code availability

- snRNA-seq data generated in this study are publicly available as of the date of publication on NCBI GEO data repository (GEO: GSE271421) and CELL x GENE repository (https://cellxgene.cziscience.com/collections/c450e15d-321a-42d6-986b-11409d04896d). Other datasets reported in this paper are publicly available as of the date of publication on the Harvard Data-verse (https://dataverse.harvard.edu/dataverse/2024_hochbaum_thyroid)
- original code reported in this paper are publicly available as of the date of publication on GitHub (https://github.com/bernardosabatinilab/hochbaum_thyroid_2024)
- Any additional information required to reanalyze the data reported in this paper is available from the lead contact upon request.

### EXPERIMENTAL MODEL AND STUDY PARTICIPANT DETAILS

#### Animals

The following mice were used in this study: C57BL/6J (Jackson labs #000664), and *Robo3*^*fl/fl*^ (gift from Alain Chédotal)^111^ bred on a C57BL/6J genetic background. Mice were maintained on a 12 h light/12 h dark cycle at a temperature of 22 ± 1 °C, a humidity of 30%–70%, with *ad libitum* access to food and water, unless on water restriction, described below. Mice were 7 weeks or older at the time of experiments and age matched across conditions, detailed below. Prior studies have implicated thyroid hormone in sexually dimorphic behaviors in many species.^17,20–29^ As we observed no statistically significant change in behavior in the light-dark assay in female mice, we performed other analyses with male mice. All animal care and experimental manipulations were performed in accordance with protocols approved by the Harvard Standing Committee on Animal Care, following guidelines described in the US NIH Guide for the Care and Use of Laboratory Animals.

### METHOD DETAILS

#### T3, sobetirome, and PTU treatments

Stock T3 (Sigma, T6397) solution was dissolved at 10 mg/mL in 100 mM NaOH and stored at −80° C. T3 working solution was prepared by making a 200x dilution of the stock in a 0.5% tween-20 solution in PBS for a final concentration of 50 μg/mL. A matching volume of 100 mM HCL was added to balance pH. The control solution was prepared identically substituting stock T3 with 100 mM NaOH, diluted 200x into the vehicle (0.5% tween-20 solution in PBS). Animals were weighed and appropriate volumes were delivered by twice-daily intraperitoneal (IP; morning and evening) injection to achieve the desired concentration. A concentration of 0.125 μg/g was used unless otherwise noted.

The vehicle solution for sobetirome^52^ (from Thomas Scanlan) experiments was made by combining Kolliphor (Sigma, C5135), NMP (Sigma, 328634), and water in a 1:1:8 ratio (KNH). Stock sobetirome solution was made by dissolving sobetirome in KNH at a final concentration of 3 mg/mL and stored at −80 °C. Sobetirome was diluted 100x into KNH for working solution and delivered by twice-daily subcutaneous injection at 0.1 μg/g unless otherwise noted. The control solution was the vehicle KNH.

Mice were given at least 2 days of vehicle control injections for habituation. Mice were then randomly assigned to cohorts injected either with the experimental solution or control solution twice per day. Experiments for the analysis of RNA were performed on the third day of treatment, after morning injection, unless otherwise noted. Experiments for the analysis of protein, electrophysiology, and behavior were performed on the fourth day of treatment, after morning injection, unless otherwise noted. For indirect calorimetry experiments of T3, treatment continued for 5 or more days (Figures 1C and S1G–S1I). For the 2ABT and physiological controls (Figures 5, 6, and S6G–S6Y), treatment continued for 8 days total (days 0–7 of treatment).

For PTU treatment, mice were transitioned to an iodine deficient diet containing PTU (0.15% PTU; Inotiv, TD.95125) or a control diet (Inotiv, TD.97350) for 3.5 weeks (randomly assigned). Although PTU mice received no injection, mice were handled twice a day for five days to provide similar habituation. On the sixth day of handling, mice were tested in the light-dark paradigm, and afterwards tissue was collected for RNA analysis.

#### Quantitative PCR

Animals were anesthetized by isoflurane inhalation and trans-cardially perfused with an ice cold choline solution containing (in mM) 25 sodium bicarbonate, 12 glucose, 1.25 sodium phosphate monobasic monohydrate, 7.5 magnesium chloride hexahydrate, 2.5 potassium chloride, 10 HEPES, 110 choline chloride, 11.6 sodium L-ascorbate, 3 sodium pyruvate, pH 7.4. Dorsal anterior cortex and liver tissues were dissected from animals and stored at −80 °C. Samples were suspended and frozen in Trizol (Life Technologies, 15596018) before RNA was extracted following the RNEasy Micro Kit (Qiagen, 74004) protocol. RNA concentrations were measured by a Nanodrop spectrophotometer (ThermoFisher, ND-2000), and samples were diluted to a 50 ng/μl RNA concentration. cDNA was generated from 1 μl of diluted RNA sample using SuperScript IV VILO Master Mix Kit with ezDNase enzyme (ThermoFisher, 11766050). Quantitative PCR was performed with TaqMan probes for target genes *Hr* (ThermoFisher, Mm00498963_m1), *Dio1* (ThermoFisher, Mm00839358_m1), *Cyp11a1* (ThermoFisher, Mm00490735_m1), and *Ier5* (ThermoFisher, Mm01295615_s1) using a standard protocol on a QuantStudio 3 (ThermoFisher, A28567). *Gapdh* (ThermoFisher, Mm99999915_g1) served as a normalization factor for all gene probes. A standard curve using ten-fold dilutions was generated for both the gene-of-interest and *Gapdh* samples. Gene-of-interest C_t_ values were normalized to their respective *Gapdh* counterparts.

#### FISH

Fresh-frozen mouse brains (11-week-old mice) were cryosectioned on a LEICA CM3050 S cryostat into 15μm sections, mounted on glass slides, allowed to dry at −20 °C for >20 minutes, and stored at −80 °C. 3- or 4-plex Fluorescent RNA in situ hybridization (FISH) was performed using Advanced Cell Diagnostics (ACD) RNAscope Multiplex Fluorescent Reagent Kit v1 (discontinued) or v2 (#323100 and #323120). Sections were prepared, pretreated, and processed according to ACD protocol except for the protease treatment, in which sections were treated with protease III (ACD, #322337) for 10 minutes at room temperature. Target probes included *Hr* (ACD, #883311), *Ier5* (ACD, #530401-C3), and *Robo3* (ACD, #558811-C2). The fluorophores used were Opal 520 reagent (Akoya Biosciences, #OP-001001), Opal 570 reagent (Akoya Biosciences, #OP-001003), Opal 620 reagent (Akoya Biosciences, #OP-001004), and Opal 690 reagent (Akoya Biosciences, #OP-001006). Nuclei were stained using the ACD supplied DAPI stain for 30 s at RT and the slides were mounted using Fluromount-G (SouthernBiotech, #0100–01). ACD’s 4-plex positive (ACD, #321811) and negative control (ACD, #321831) probes were used for experimental signal verification.

Samples were imaged on a Leica SP8 X confocal microscope with a 63×, 1.4-NA oil-immersion objective (Harvard NeuroDiscovery Center). We imaged areas of M2 that contained all cortical layers, with optical sectioning of 0.5 μm. For analysis, we utilized machine learning software^115^ to segment DAPI-stained nuclei and to count the fluorescent puncta of hybridized probes. For large area image presentation (Figures 1B, 2G, and S1F), nuclei masks created in the analysis pipeline are displayed and pseudocolored according to the number of puncta contained within the mask.

#### Indirect calorimetry

14-week-old C57BL/6J male mice were housed at 23 °C in a Promethion indirect calorimetry system (Sable Systems International) within a temperature-controlled cabinet. The mice were injected with vehicle, T3, or sobetirome as described above. Data collected include VO2, VCO2, physical activity beam breaks, food intake, and body mass. For the duration of the experiment, the mice had ad libitum access to Labdiet 5008 (3.56 kcal/g) and were maintained on a 12hr/12hr photoperiod with lights on from 0600/1800.

#### Light-dark behavioral paradigm

The Light-Dark box arena (27.3cm × 27.3cm × 20.3cm, Med Associates, ENV-510S-A) was divided into two compartments: an uncovered area under direct light and a covered area separated with an opaque plexiglass structure (Med Associates, ENV-511). An opening (6cm × 5 cm) in the plexiglass separator allowed mice to move between the two compartments. Mice (11–12 weeks) spent 10 minutes in the arena. Time spent in the light and dark areas was analyzed using the Activity Monitor software (Med Associates, version 7). Light-dark boxes were cleaned with ethanol before each mouse began its trial and allowed to dry. Animals were 11–12 weeks old at the time of the experiment. In testing dilutions of T3 (Figure S2D), experiments consisted of multiple rounds over many months and by necessity not every T3 dilution was contained within each round. The data from each round was normalized to the control cohort of mice (median of 4 or more control mice) in the same round to account for round-to-round variability and allow for robust comparison across all dilutions.

#### Open-field paradigm

Mice (11–12 weeks) were placed into an arena (same as light-dark assay, without the insert: 27.3cm × 27.3cm × 20.3cm, Med Associates, ENV-510s-A) and monitored for 60 minutes. The arena was cleaned with ethanol before each mouse began its trial and allowed to dry. Locomotor activity was analyzed using the Activity Monitor software (Med Associates, version 7). Mice tested in this paradigm were tested after 4.5 days of treatment. On the previous day (3.5 days of treatment) these mice were tested in the light-dark assay described above.

#### MoSeq open field recording

The depth video recording apparatus and open-field arena (OFA) were prepared according to the Motion Sequencing (MoSeq) protocol.^54^ The OFA was an opaque circular plastic bucket with 14”-high walls and a 17” diameter (US Plastics 14317). Mice (n=12 T3 treatment, n=14 control) were habituated to the behavioral room and OFA for 3 days. On each habituation day, the mice were brought to the behavior room to habituate in their home cages for a minimum of 1 hour with lights off. The mice were then placed in the OFA to freely explore for 10 mins. The order of the mice going into the arena was randomized on each day. Between mice, the arena was cleaned according to the MoSeq protocol.^54^ Following the habituation days, mice were treated with T3 or vehicle control as described above and recorded for 4 days (recordings occurred ~5 hours after morning treatment). During the behavioral depth recording sessions, the order of mice was randomized, and each mouse was placed in the OFA to freely behave for 30 minutes. The behavioral depth videos were recorded at 30Hz with a Microsoft Kinect 2 depth sensor. The mice were returned to their home cages after the recordings and the OFA was cleaned.

#### Immunohistochemistry

Animals were anesthetized by isoflurane inhalation and trans-cardially perfused with cold 0.9% saline followed by 4% paraformaldehyde (PFA) in a phosphate buffered solution (PB) (0.081 mM Na_2_HPO_4_, 0.017 mM NaH_2_PO_4_, pH 7.2–7.4). Brains were harvested and post-fixed in 4% PFA overnight at 4C and preserved in 0.5xPB + 0.02% sodium azide. Brains were sliced coronally at 50 μm and slices were permeabilized with a blocking buffer containing 0.1% Triton-X, 6% normal goat serum (NGS, Abcam ab7481) in PBS for 2 hours at RT. Primary antibodies were applied at 4° C overnight. Secondary antibodies were applied for 2 hours at room temperature and counterstained with DAPI (Sigma Aldrich D9542, 1:10000) for 20 minutes. Slices were then mounted to glass slides and cover-slipped with Vectashield Vibrance (Vector H-1700) Primary antibodies used for immunohistochemistry (IHC) are listed here with dilutions indicated in parentheses: rat anti-HA (Roche 11867423001, 1:500), chicken anti-GFP (Abcam ab13970 1:1500), rabbit anti-NeuN (Sigma Aldrich ABN78). Fluorophore-conjugated secondary antibodies for IHC: goat anti-rat Alexa 555 (ThermoFisher A21434, 1:500), goat anti-chicken Alexa 488 (ThermoFisher A11039, 1:500), goat anti-mouse Alexa 647 (ThermoFisher A21235, 1:500), streptavidin Alexa 647 (ThermoFisher S32357, 1:50). Samples were imaged on a VS200 Slide Scanner (Olympus) using a 10x objective lens (Figures 3C, 3D, and S6A–S6D) or on a Leica SP8 X confocal microscope with a 63×, 1.4-NA oil-immersion objective (Harvard NeuroDiscovery Center, Figure 4B).

#### Robo3 protein extraction

*Robo3*^*fl/fl*^ mice were retro-orbitally injected with a systemic AAV serotype (PHP.eB) capable of efficiently transducing a Cre transgene to neurons in cortex (pENN.AAV_PHPeB.hSyn.HI.eGFP-Cre.WPRE.SV40, ~5–10 × 10^11^ vg). We then waited ~3 weeks to allow for Cre-mediated excision of *Robo3* and subsequent protein turnover. Animals (C57BL/6J, un-transduced *Robo3*^*fl/fl*^, and Cre-transduced *Robo3*^*fl/fl*^) were treated with T3 or vehicle control. After 3.5 days of treatment frontal cortex was dissected and submerged in ice-cold 300 μl of RIPA buffer (ThermoFisher, #89900) supplemented with protease inhibitors (cOmplete mini, Roche, #11836170001). The tissue was lysed in a Dounce homogenizer with 40 strokes of a plunger, and the resultant lysate was incubated with end-over-end rotation for 10 minutes at 4°C. The lysate was then cleared by centrifugation (17000g, 10 minutes, 4°C), and the supernatant was passed through a Wizard column to remove genomic DNA (Promega) (10000g, 1 minute, 4°C). Protein from lysates were denatured by the addition of 50 ml of Laemmli sample buffer (37 mM Tris-HCl, pH 6.8, 10% (wt/vol) SDS, 25% (vol/vol) 2-mercaptoethanol, 25% (vol/vol) glycerol, and 0.056% (wt/vol) bromophenol blue). Samples were resolved by 8–16% SDS-PAGE, transferred for 2 hours at room temperature at 45 V to 0.45 mm PVDF membranes, and analyzed by immunoblotting. Membranes were blocked with 5% milk prepared in TBST (Tris-buffered Saline with Tween 20) for at least 5 min at room temperature, then incubated with primary antibodies in 5% BSA TBST overnight at 4°C with end-over-end rotation. Primary antibodies targeting the following proteins were used at the indicated dilutions and obtained from the denoted companies: Robo3 1:300 (R&D systems, #AF3155), GluN1 NMDAR 1:1000 (SySy #114011). Following overnight incubation, membranes were washed three times, 5 min each, with TBST and incubated with the corresponding secondary antibodies in 5% milk (1:5000) for 1 hour at room temperature. Membranes were then washed three more times, 5 min each, with TBST before being visualized using enhanced chemiluminescence (ThermoFisher, #32106). Signals were quantified using ImageJ (Fiji).^112^

#### Generation of single-nucleus suspensions from mouse secondary motor cortex

Fresh-frozen mouse brains (10-week-old mice) were securely mounted by the cerebellum onto cryostat chucks using OCT embedding compound without thermal perturbation. Bilateral dissection of the anterior regions of M2 was performed by hand in the cryostat using an ophthalmic microscalpel (FEATHER Incision scalpel P-715) and 4x surgical loupes. Each dissected sample was placed into a 0.25 mL PCR tube using forceps. All materials were pre-cooled to −20° prior to use.

Nuclei isolation was then performed as previously described.^116,117^ Briefly, sectioned tissues were moved from the cryostat into 12-well plates (one well per sample), and 2 mL of extraction buffer was added to each well. Mechanical dissociation was performed by slowly triturating up and down 20 times using a P1000 pipette (1 mL Rainin tip), with extra care to avoid froth or bubbles. This trituration step was repeated three or four additional times, with a 2 minute break between rounds. Using a syringe, we passed each sample twice through a 26-gauge needle into its original well. ~2 mL of this solution was transferred into a 50 mL conical tube for each sample, followed by the addition of wash buffer to a total volume of ~20 mL. This mixture was then split equally into two 50 mL conical tubes. The samples were spun down in a swinging-bucket centrifuge at 600 RCF for 10 min at 4°C. After centrifugation, the supernatant was carefully removed, making sure not to disturb the pellet, until ~500 μL remained in the tube. The resuspended nuclei were then pooled back into a single tube, resulting in ~1 mL of concentrated nuclei solution. DAPI (Thermo Fisher Scientific, no. 62248) was added at 1:1000 concentration and incubated for at least 5 minutes prior to sorting. Singlet nuclei were isolated utilizing fluorescence activated cell sorting on a Sony SH800, and the final nuclei concentration was determined using a hemocytometer.

#### snRNA-seq library preparation and sequencing

The 10X Genomics 3’ v3 kit was used for library preparation according to the manufacturer’s recommendation. Libraries were pooled and sequenced on either a NovaSeq S2 or NovaSeq S4. Using Cell Ranger v5, sequencing reads were demultiplexed, aligned to the GRCm38 reference genome, which was custom annotated to facilitate readout of Cre and DN/WT-THR transgene expression, and filtered for valid 10x barcodes, UMI correction, and cell-calling.

#### snRNA-seq data preprocessing

Filtered gene expression counts matrices were loaded into R and converted to Seurat objects. Sequencing replicates were merged and a first round of QC was performed to remove nuclei with fewer than 200 unique genes (nGenes) detected and greater than 5% mitochondrial genes (MT%). The data were annotated to reflect the experimental “genotype” (“endogenous” meaning non-transduced C57BL/6J mice, “DN-THR” meaning virally transduced C57BL/6J with Cre and Cre-dependent DN-THR, or “WT-THR” meaning virally transduced C57BL/6J with Cre and Cre-dependent WT-THR) and treatment condition (T3 or vehicle control). Additional quality control metrics, including number of unique molecular identifiers (nUMIs), percent oxidative phosphorylation genes (% OXPHOS), and percent ribosomal protein–were calculated and gene names were mapped to Ensembl gene IDs. The virally transduced Cre and DN- or WT-THR transcripts were annotated prior to removal of any other genes lacking Ensembl gene IDs or with duplicate Ensembl gene IDs.

#### Clustering and major cell class annotation

Highly variable gene selection was performed on log-normalized counts data using Seurat’s vst method to identify the top 2000 genes with expression patterns that drive biological differences between cell types while minimizing batch effects. After applying ScaleData() to set the mean expression of each variable gene to 0 and variance across cells to 1, we performed preliminary principal component analysis (PCA; k=25) and Uniform Manifold Approximation and Projection (UMAP) dimensionality reduction. Louvain clustering was used throughout all analyses with clustering resolutions of 0.6 or 1.0. Clusters with high MT% and OXPHOS%, which also had no clearly delineated cell type markers, were identified and removed from downstream analyses. A second round of PCA (k=100) and UMAP was performed on the filtered dataset. Resulting clusters were annotated into one of seven major cell classes (glutamatergic neurons, GABAergic neurons, astrocytes, oligodendrocytes, oligodendrocyte precursor cells, immune cells, and endothelial cells) based on per-cluster expression of a list of marker genes.^55^ Putative doublets (clusters that showed substantial expression of marker genes from two or more major cell types) were removed.

#### Subtype annotation based on mouse motor cortex reference dataset

Subtypes within each broad cell class were assigned using a random walk algorithm^113^ to transfer annotations from a recently published transcriptomic atlas of the mouse motor cortex^55^ (the “source” dataset) to our study dataset (the “target” dataset). For glutamatergic and GABAergic neurons, the original embedding space was sufficient for obtaining a high-quality annotation of clustered subtypes due to limited sample-to-sample batch effects and high similarity to the source dataset. For glial cell types (except oligodendrocyte precursor cells for which there was only one cluster), each cell class specific subset of our target dataset was first aligned to the corresponding cell types of the source dataset by performing an integrative analysis including highly variable gene selection and PCA (k=30 for oligodendrocytes and endothelial cell classes; k=100 for astrocytes and immune cell classes). We utilized Harmony to account for batch effects between the source and target datasets and to flag additional doublet clusters revealed by this cell class specific process for removal. Several immune cell clusters from the DN- and WT-THR genotypes that highly expressed the inflammatory marker *Cxcl10*, failed to align with the source dataset, most likely due to a reactive microglia response to viral infection. 10,807 (49.2%) of all immune cells were thus removed from subsequent DE analyses, of which 10,675 (98.8%) had DN- or WT-THR genotypes. Ultimately, we analyzed a filtered dataset of 107,742 cells in the endogenous cohort and 205,772 DN-THR or WT-THR cells. Less than 1.5% of all cells analyzed had fewer than 1000 nUMIs and the minimum nGenes was 498. Distributions of quality metrics per sample are included in Figure S4A. Subtype annotation was verified with a per-cluster marker analysis visualized in Figure S4B.

#### Stereotaxic surgeries

Mice were anesthetized with 2%–3% isoflurane and 0.08% oxygen, and surgeries were performed under aseptic conditions within a stereotaxic frame (David Kopf Instruments). For intracranial injections, small craniotomies were drilled using a #81 drill bit (Kyocera, 20–517-025) and injections were performed through a pulled glass pipette containing virus, driven by a syringe pump (Harvard Apparatus, #883015) at a rate of 40 nL/min. After injections, the wound was sutured, mice were placed in a cage with a heating pad until their activity recovered, and then they were returned to their homecage and were given pre- and post-operative oral carprofen (CPG, 5 mg/kg/day) and monitored daily for at least 4 days post-surgery. Experiments were performed at least 13 days after viral injections to allow for transgene expression.

For 2ABT experimental animals, the same surgical setup was used to install a headpost. Briefly, the skull was lightly scored with a scalpel and a metal headpost was glued at lambda (Loctite gel #454). White cement (Flow-It ALC) was used to secure a border between the skin and the skull. For animals included in 2ABT experiments described in Figure 6, a drill bit was used to lightly mark coordinates for future viral injections. The remaining exposed skull was covered with silicon (KwickKast). Animals were given CPG (5 mg/kg/day) and monitored as above before beginning 2ABT training.

The following coordinates were used as injection sites (relative to bregma):

Figures 3 and S4G–S4I: +2.4 mm and +1.8 mm A/P, +/−1.0 mm M/L, and 0.6 mm D/V.

Figures 4 and S5C–S5R: +2.2 mm A/P, +/−1.0 mm M/L, and 0.4 mm D/V.

Figures 6, S6A–S6F, and S6M–S6Y: +2.5mm A/P, +/− 1.5mm and +/− 0.5mm M/L, 0.4 and 0.9 mm D/V.

The following viruses were used for injections (approximate final titer in gc/ml):

Figures 3 and S4G–S4I: 400 nL scAAV2/9-hSyn-iCre-HA (9 × 10^10^) combined with either AAV2/9-SIO-nEF-DN-Thrb (5 × 10^12^) or AAV2/9-SIO-nEF-WT-Thrb (5 × 10^12^).

Figure 4: Left hemisphere: 200 nL AAV2/9-hSyn-FLEX-CoChR-GFP (5 × 10^12^). Right hemisphere: 200 nL AAV2/retro-CAG-Cre (5 × 10^12^).

Figure S5C: Left hemisphere: 250 nL AAV2/9-hSyn-CoChR-GFP (1 × 10^12^). Right hemisphere: no virus.

Figures S5D–S5F: Both hemispheres: 250 nL AAV-S5E2-ChR2-mCherry (1 × 10^13^).

Figures S5G–S5L: Left hemisphere: 200 nL AAV2/9-hSyn-FLEX-CoChR-GFP (1 × 10^12^) combined with either AAV2/9-SIO-nEF-DN-Thrb (4 × 10^12^) or AAV2/9-SIO-nEF-WT-Thrb (4 × 10^12^). Right hemisphere: 200 nL AAV2/retro-CAG-Cre (5 × 10^12^).

Figures S5M–S5R: Left hemisphere: 200 nL AAV2/9-hSyn-CoChR-GFP (1 × 10^12^) combined with either scAAV2/9-hSyn-iCre-HA (1 × 10^12^) or scAAV2/9-hSyn-ΔiCre-HA (1 × 10^12^). Right hemisphere: no virus.

Figures 6, S6A–S6F, and S6M–S6Y: 200 nL AAV2/9-hSyn-DN-THR (1×10^13^), or 200 nL AAV2/9-hSyn-WT-THR (1×10^13^).

#### Electrophysiology

Brain slices were obtained from 2.5- to 4.5-month-old mice using standard techniques. Mice were anaesthetized by isoflurane inhalation and perfused trans-cardially with ice-cold ACSF containing (in mM) 125 NaCl, 2.5 KCl, 25 NaHCO_3_, 2 CaCl_2_, 1 MgCl_2_, 1.25 NaH_2_PO_4_, and 11 glucose (295 mOsm/kg). Brains were blocked, cut along the midline, and hemispheres with contralateral projecting CoChR+ axons were transferred into a slicing chamber containing ice-cold ACSF. Coronal slices of frontal cortex were cut at 300 μm thickness with a Leica VT1000 S vibratome in ice-cold ACSF, transferred for 10 min to a holding chamber containing choline-based solution (consisting of (in mM): 110 choline chloride, 25 NaHCO_3_, 2.5 KCl, 7 MgCl_2_, 0.5 CaCl_2_, 1.25 NaH_2_PO_4_, 25 glucose, 11.6 ascorbic acid, and 3.1 pyruvic acid) at 34°C then transferred to a secondary holding chamber containing ACSF and maintained at room temperature (20–22°C) until use. All recordings were obtained within 6 hours of slicing. Both choline solution and ACSF were bubbled with 95% O2/5% CO2.

Acute coronal slices were maintained in ACSF at 34 °C during recordings. Whole-cell recordings were obtained from L2/3 glutamatergic neurons (150–350 μm below the pia surface) at ~2.5–2 mm anterior to bregma, and ~1 mm lateral of the midline identified by morphological (pyramidal cell body) and electrophysiological (membrane capacitance C_m_>75 pF, membrane resistance R_m_<300 MΩ) features. For recordings of optically evoked PSCs, pipettes were pulled to have a resistance of 2–3 MΩ and were filled with internal solution containing (in mM), 135 CsMeSO_3_, 10 HEPES, 1 EGTA, 3.3 QX314-Cl, 4 Mg-ATP, 0.3 Na-GTP, 8 Na_2_-phosphocreatine. In a subset of experiments, 1 mg/ml biocytin (Sigma B4261) was added for post-hoc staining. Whole-cell recordings were conducted in voltage-clamp mode, first at −70 mV to record evoked EPSCs, then at 0 mV to record evoked IPSCs. Full field illumination (0.13 mm^2^) was delivered by a 473 nm laser whose power output was controlled through an acoustooptic modulator. A 2 ms light pulse was delivered at randomized powers (7–10 discrete powers delivered between ~0.03 to 4 mW), with 15 seconds between pulses to allow for recovery. Recordings of currents during each trial were 3 s long, with a 1 s baseline prior to light pulse delivery. Each light power was repeated 3 times. For measurements of optical paired pulse ratio (PPR), powers in the above range were tested to find the lowest power producing an EPSC of ~100–500 pA. Light pulses were either delivered alone as above, or two were delivered 50 ms apart to measure isolated, and paired EPSCs. Stimuli order was randomized, and each stimulus was repeated 5 times, and 10 μM Gabazine (SR 95531 hydrobromide, Tocris 1262) was included the ACSF. There was no difference in initial EPSC size between control and T3 treated samples (*p*=0.6, Wilcoxon rank-sum test) and PPR did not scale with initial EPSC size (linear regression, F=0.465 (1,26), p=0.50). For experiments stimulating channelrhodopsin-labeled PV neurons, light powers were reduced to ~0.01 to 1 mW, and 10 μM NBQX (Tocris 1044) and 10 μM CPP (Tocris 0247) were added to the ACSF.

Quality control criteria were 1) manual inspection of unlabeled recordings for baseline stability (blinded to condition), 2) generation of peak currents > 250 pA at saturating light stimuli (> 3 mW for trans-hemispheric axon stimulation, > 0.5 mW for local PV neuron stimulation, N/A for PPR) to indicate that recordings were made within the channelrhodopsin-labeled axonal field, 3) variation in Rm < 20% between recordings 4) holding current I_h_ < −400 pA at V = −70 mV, and 5) variation in series resistance Rs < 25% between recordings and V = −70 mV and V = 0 mV recording epochs. For experiments with recordings at only one holding potential a hard threshold of Rs < 25 Mohm was used in lieu of the stability criteria.

For measurements of intrinsic excitability, L2/3 glutamatergic neurons were targeted as above. Pipettes were filled with a potassium-based internal solution containing (in mM), 135 KMeSO_3_, 3 KCl, 10 HEPES, 1 EGTA, 0.1 CaCl_2_, 4 Mg-ATP, 0.3 Na-GTP, 8 Na_2_-phosphocreatine. Recordings were made in current-clamp configuration. Resting membrane potential (RMP) was first recorded, and then adjusted to −80 mV. There was no difference in recorded RMP between T3 and C (−87 ± 4 mV, −85 ± 5 mV, p=0.06, Wilcoxon rank-sum test). 1 s current injections were delivered in 50 pA increments between −100 to 650 pA in random order., with an inter-pulse interval of 10 s. Recordings of each current injection were 2.5 s long, with a 1 s baseline prior to current injection. Experiments were repeated using alternate divalent cation concentrations in the ACSF, identical to the above except 1 mM CaCl_2_ and 2 mM MgCl_2_. Quality control criteria were a stable baseline membrane resistance (variation in R_m_ < 10%) and a stable baseline resting potential with median standard deviation of ≤ 0.5 mV across recordings. For all electrophysiology, the experimenter was blinded to treatment condition.

#### 2ABT

Mice had a headpost installed as described above. After recovery, mice were water restricted to 1 mL per day for five days prior to training. Mice were maintained at >80% initial body weight for the full duration of the training. Mice were age 7–11 weeks at the start of water restriction.

The behavior apparatus was contained within a sound-attenuating box (Med Associates ENV-018V) and consisted of a custom-built two-tiered platform. Two steel spouts (0.05” OD, 0.033” ID), 0.5 cm apart, were mounted on a 2-axis motor stage (Zaber A-MCB2-KS10A) via a post equipped with a manual 3-axis fine positioning stage (Narishige U-3C). Animals were located on the top tier of the platform on a detachable stage. The stage consisted of a copper lined, clear, polycarbonate round tube (3.8 cm ID × 10 cm, McMaster-Carr) with a 2 cm long, 3/4 cylinder opening, fixed to a circular aluminum breadboard (15 cm × 0.127 cm, Thorlabs MBR6) via an adjustable-height optics clamp (Thorlabs VG100). Custom metal headpost holders were secured to the stage, directly flanking the small opening of the tube, using two hex-locking post holders (2.5 cm, Thorlabs PH1) and mounting bases (2.5 cm × 5.8 cm × 1 cm, Thorlabs BA1s). The stage was secured within the behavior apparatus using kinematic bases with magnetically coupled plates (7.6 cm × 7.6 cm, Newport BK-3A). Speakers (Audax TW025A20) were mounted on the lower tier of the platform to provide auditory cues. Solenoids (Lee LHQA0531220H) were used to deliver water droplets.

The behavior task was run using custom software (LabVIEW 2014, National Instruments) utilizing a MyRio-1900 (National Instruments). The trial structure consisted of an enforced no-lick period (1–2 s), in which any lick resulted in a trial restart, and a tone (75 ms long, 5 khz) marking the beginning of a selection period (3 s). During this period, a mouse could make a choice by licking one of the two spouts. Probability of water delivery was assigned in software. If successful, 2.5 ul of water was delivered from the selected spout during a 3 s consumption period (which occurred regardless of outcome). The trial was terminated and the outcome was a timeout if no choice was made during the selection period. Trials were delineated into blocks in which one spout has a higher probability of reward than the other. At the end of each block period, the reward probabilities reversed.

Mice were first trained on a deterministic version of the task to teach switching between spouts. For each trial, one of the two spouts delivered a water droplet. The block length in the deterministic task was 8–9 rewards. Spout probabilities reversed after mice received the allotted number of rewards. After 3–7 days, mice were introduced to a probabilistic task with a block length of 20 trials. One spout within a block had a 90% probability of delivering a water droplet while the alternate spout had a 10% probability (90/10). These probabilities reversed with each block. After 1–8 more days of training, the reward probabilities were changed to 80% for one spout and 20% for the alternate spout within a block (80/20). For the cohort of wildtype animals (Figures 5C–5J and S6G–S6L) this was the final task structure. For the cohort of WT-THR or DN-THR injected animals (Figures 6 and S6R–S6Y) we altered the block structure such that the block length varied randomly from 20–40 trials with an approximate exponential distribution (43% probability of a block of 20 trials in length, 32% probability of a block of 30 trials in length, and 25% probability of a block of 40 trials in length).

Performance was evaluated by probability of selecting the highly rewarding spout (p(high)) averaged over 5 days of training. Performance criterion was met by achieving a 5-day window with average p(high) > 0.6. The cohort of wildtype animals (Figures 5C–5J and S6G–S6L) began experiments once meeting performance criterion and having trained at 80/20 for 10 or more days. Mice were treated with vehicle solution (described above) for 3–5 days to establish baseline behavior (habituation sessions), and then were treated with T3 or continued vehicle administration for 8 days (days 0–7 of treatment). The cohort of WT-THR or DN-THR animals (Figures 6 and S6R–S6Y) were given free access to water after reaching performance criterion at 80/20, and intracranial injections of AAVs encoding either WT-THR or DN-THR were performed (described above, AAV type was alternately assigned to ensure both cohorts had similar experimental timeframes). After post-operative recovery, mice were water restricted again and reintroduced to the task. After at least 13 days post-surgery, to allow for WT- or DN-THR expression, mice began the experiment, with 4–6 habituation sessions followed by 8 days of T3 treatment (days 0–7 of treatment). After completion of the final day of the task, most mice were perfused as described above (immunohistochemistry) for brain-wide mapping of DN-THR or WT-THR (Figures 6A, 6B, and S6M). DN-THR and WT-THR constructs have a C-terminal HA-tag to enable quantification using immunohistochemistry and imaging. Whole brain 50-micron slices were stained for the HA-tag and DAPI (described above) and were mounted and imaged using an Olympus VS200 Slide Scanner.

For analyses comparing before and after treatment, habituation sessions were compared to sessions from days 4–7 of treatment from individual mice. For mice with more than 4 habituation sessions, the last 4 habituation sessions were used to ensure the datasets were balanced. The first block of trials, as the animals were reacquainted with the task, was not included in analyses. Timeout trials (rare in trained animals: 0.025 ± 0.02 of trials, mean ± SD) were not included in analyses and modeling of mouse choice. When possible, experimenters that trained animals were different than those that performed viral injections of WT-/DN-THR to enable blinded experiments in a subset of mice.

#### Thirst measurements

Mice were injected with AAVs expressing either DN-THR or WT-THR in frontal cortex as described above (same as DN-/WT-THR 2ABT mice; Figures 6 and S6R–S6Y). After recovery, the water intake of single-housed animals was monitored using a 15 ml glass bottle (Amuza) and leak resistant sipper tube (65 mm, Amuza). During the experiment, mice were treated with vehicle solution (habituation, described above) for 5 days to establish baseline water intake, and then treated with 8 days of T3 treatment (day 0–7 of treatment). Water intake was measured and recorded every day at the same time (6 pm) throughout the experiment.

#### Heart rate measurements

Mice were injected with AAVs expressing DN-THR in frontal cortex as described above (same as DN-/WT-THR 2ABT mice; Figures 6 and S6R–S6Y). After recovery, a plastic training collar was placed dorsally around the neck of DN-THR expressing mice or age-matched un-injected control mice to allow them to habituate to the collar for one hour in their home cage. The following day, a pulse oximetry sensor collar (MOX-SNSR-CLLR-S, Starr Life Sciences Corporation) was fitted to the mouse. Mice were placed in a clear, conscious measurement animal enclosure (MOX-STCME-M, Starr Life Sciences Corp) with fresh bedding and connected to the MouseOx Plus system. Mice were habituated to the chamber for 15 minutes or until heart rate recordings stabilized and the mouse appeared calm. Animals began experiments the following day. Mice were treated with vehicle solution (habituation, described above) for 5–7 days to establish baseline heart rate, and then treated with 8 days of T3 treatment (day 0–7 of treatment). After settling into the chamber (~3 min), stable heart rate recordings of > 20 seconds were captured using the MouseOx Plus Conscious Applications Module software. Heart rate (beats per minute) was sampled at 15 Hz. Data was thresholded at 500 bpm to account for occasional missed detection, likely due to movement artifact. Measurements were performed daily in control and DN-THR mice over the course of habituation and treatment, and median heart rate was calculated from each recording.

### QUANTIFICATION AND STATISTICAL ANALYSIS

#### MoSeq behavior analysis

The recorded mouse depth videos (i.e., sessions) were processed and modeled using the MoSeq pipeline available at http://www.moseq4all.org/. MoSeq is an unsupervised machine learning algorithm that parses animal spontaneous behavior into sub-second, repeated, modulated motifs called “syllables”. The pipeline assigned each frame of the depth videos a syllable label then identified the number of occurrences for each syllable and the number of transitions from one syllable into another syllable. Only the syllables that made up more than 1% of all syllable occurrences identified across all the sessions were used in the analysis (47 syllables in total). The MoSeq pipeline output kinematic values such as mouse centroid coordinates, speed and distance from mouse centroid to arena centroid for each frame. Syllable usages were computed from the percentage of syllable instances within a session. The normalized position is the ratio of distance to arena centroid to OFA radius. The heatmaps for MoSeq syllable usages, speed and position are binned percentages scaled by a Min-Max scalar. The total distance traveled in a session is the sum of the between-frame Euclidean distance between the mouse centroid coordinates for each pair of consecutive frames. Syllables with significantly different usage between the treatment group and the control group were identified using Kruskal–Wallis test and a post hoc Dunn’s two-sided test with permutation, using Benjamini–Hochberg FDR (α = 0.1), described in detail in Wiltschko et al.^118^ The usage entropy of each session is the Shannon entropy over the syllable usage distribution within the session. The outgoing entropy for each syllable within each session is the Shannon entropy over the distribution of outgoing transitions (i.e., one syllable transitioning into another) for the syllable in that session. The outgoing entropy of each session is the mean of all the syllable outgoing entropies within the session.

#### Pseudocell-based differential expression analysis

We utilized a previously developed pseudocell strategy^113^ coupled with linear modeling to identify differentially expressed (DE) genes. Within each cell subtype, we constructed pseudocells by aggregating the raw UMI count of, on average, 30 nuclei per sample. Each resulting pseudocell was thus composed of randomly sampled (rand_pseudobulk_mod=T) nuclei from mice of the same genotype (endogenous, DN-THR, or WT-THR) and treatment status (T3 or vehicle control). Pseudocells had a minimum of 15 nuclei, and cell subtypes with fewer than 15 nuclei or 6 total constructed pseudocells were excluded from analysis.

For all comparisons, we implemented the Limma Trend approach with robust moderated t-statistic and Benjamini-Hochberg FDR-adjusted *p* values to identify DE genes for each cell subtype. %MT and log_2_(nGene), calculated at the pseudocell level, were used as covariates and sample ID was used as a random effect where applicable. Genes expressed in greater than one percent of cells of the relevant subtype were used as background for the differential expression testing.

We used the above DE methodology to assess the effect of thyroid hormone on gene expression in the motor cortex. DE analysis was performed on pseudocells between T3- and vehicle control-treated samples. A robustness score (robScore_logFC; defined for each gene as the fraction of sample pairs in the experiment showing up- versus down-regulation based on the mean expression across the tested condition) and robustness percentage (robScore_pct; defined similarly but using percent non-zero expression) was calculated for each gene. A TRG was then defined as any DE gene with FDR-adjusted *p* value < 0.05 and |robScore_logFC| ≥ 0.5, meaning that a gene is consistently up- or down-regulated in at least 75% of comparisons. In dot plot figures (Figures 2F and S4C), dots are only shown for genes in cell types where the plotted gene passed criteria to be classified as a TRG. Non-TRGs (Figures 3I and S4I) were defined by subsetting the top 10,000 most expressed genes with FDR-adjusted *p* value > 0.05, robScore_logFC = 0, and robScore_pct = 0.

A second set of DE analyses were performed (Figures 3 and S4G–S4I) to evaluate the effect of DN-THR or WT-THR expression on the TRG programs identified above. New pseudocells were constructed by sorting nuclei according to their genotype (DN-THR or WT-THR only), treatment (T3 or vehicle control), and Cre status. Each pseudocell was composed of exclusively “Cre positive” nuclei— defined as having 1 or more detected Cre transcript(s) as a readout of the presence of activated Cre-dependent DN-THR/WT-THR constructs— or “Cre negative” nuclei— defined as having 0 detected Cre transcripts. DE analyses were performed to compare the Cre+ and Cre− conditions. Given sample size limitations for the DN-THR/WT-THR experiment (2–3 mice per genotype), robustness scores were not calculated.

#### TRG correlation matrix

To produce the correlation matrix in Figure 2D, the Spearman correlations between log_2_(fold-change) values were calculated across all TRGs for each pair of cell types. Hierarchical clustering was then performed on the Euclidean distances of this TRG correlation matrix by implementing Ward’s clustering criterion (“ward.D2”). Only cell types that induced TRGs were included in this analysis.

#### Ordered gene set enrichment analysis

For each cell subtype analyzed through the above differential expression framework, the background genes used in the DE analysis (genes expressed in >1% of cells of the relevant subtype) were annotated using the gene biotype information in the “EnsDb.Mmusculus.v79” package in R Bioconductor and filtered to include only protein-coding genes. These resulting genes were then ordered by the absolute value of the t-statistic derived from the Limma Trend model, such that the most significantly perturbed genes–regardless of the direction of the perturbation–were highest in the ranked list. The 2023 GO Biological Process gene set was obtained from the EnrichR portal^119^ and the fGSEA package v1.24.0^114^ was run with a “pos” scoreType, minimal gene set size of 15, and maximal gene set size of 500. To identify top fGSEA terms for each subtype (Figure 2E), we subsetted significantly enriched pathways (FDR-adjusted *p* value < 0.05) in which the leading-edge gene subset (i.e. the genes driving the enrichment signal) contained at least 3 genes with FDR-adjusted *p* value < 0.05, |robScore_logFC| ≥ 0.5, and non-zero counts per 100k cells log_2_(fold-change) after DE analysis. The union of the top 3 fGSEA terms, ranked by FDR-adjusted *p* value, were included for each neuronal cell subtype.

#### PSC and intrinsic excitability quantification

All analyses and figures related to electrophysiology recordings of PSCs use the 10 ms integrated charge at each laser power, normalized by $C_{m}$. These values were fit in a custom MATLAB (2021b) script to a sigmoid function, $\frac{a}{1+{\left(\frac{b}{x}\right)}^{c}}$, where *x* is the laser power, to find optimal values for coefficients. For figures, the log_2_(fold-change) value of an individual coefficient relative to the median control condition coefficient value is presented. PPR was calculated as 10 ms integrated charge of the second EPSC divided by the 10 ms integrated charge of the first EPSC. To isolate integrated charge associated with the second EPSC, the average of the EPSC produced by the single pulse stimulus was subtracted from the PPR trace. All analyses of action potential waveform and generation from current injection were performed with values directly extracted from ScanImage.^120^

#### 2ABT behavioral modeling

To model mouse choice behavior in the 2ABT we used Q-learning, a reinforcement learning framework that estimates values of each potential action decision and uses these values to generate predicted choices. In this case there are only 2 action values, one for selecting the left spout, $Q_{l}$, and the other for selecting the right spout, $Q_{r}$. These values evolve according to:

$$\text{selected}\;\text{spout}\;\text{i}\;:\;Q_{i}(t+1)=Q_{i}(t)+\alpha \left(R-Q_{i}(t)\right)\;\;\text{unselected}\;\text{spout}\;\text{j}\;:\;Q_{j\neq i}(t+1)=(1-\zeta )Q_{j\neq i}(t)$$

where R is the state of the water reward (R=1 for a dispensed water droplet, R=0 for a failure), α is the learning rate that scales the value update of the selected spout, and ζ is the forgetting rate which decrements the value of the unselected spout. Q-values are then fed into a Boltzmann distribution (softmax function) that determines the policy (probability) of selecting each spout:

$$P_{i}(t)=\frac{1}{1+e^{-[\beta (Q_{i}(t)-Q_{j}(t))+b]}}\;P_{j}(t)=1-P_{i}(t)$$

where β is an inverse temperature parameter given relative values between the spouts $Q_{i}(t)-Q_{j}(t)$ and b is a bias parameter. β scales the slope of the sigmoidal activation curve and varies the degree to which actions are stochastic and exploratory versus exploitative of current value estimates. Models were fit to individual mice for habituation sessions, and separately for sessions from days 4–7 of treatment. Each session was split into 4, and model training was performed on randomly selected 3 of the 4 blocks of trials (75%), with the remaining block used as held out data to evaluate fits (25%). Parameters β,α,ζ, b were estimated using stochastic gradient descent optimization (learning rate of 0.1, 10000 iterations) and a negative log-likelihood loss function (custom Python code using PyTorch library). All fits were evaluated on the 25% held out data (Figures 5I, S6U, and S6V).

#### Brain-wide mapping of DN-THR and WT-THR expression

DN-THR and WT-THR constructs have a C-terminal HA-tag to enable quantification via using immunohistochemistry and imaging. Whole brain 50 μm slices were stained for the HA-tag and DAPI (described above) and were mounted and imaged using an Olympus VS200 Slide Scanner. NeuroInfo (Version 2020.1.1, 64 bit) with the built-in BrainMaker Workflow was used to load and realign images of brain slices stained for the HA-tag and DAPI from the majority of DN-THR and WT-THR animals used in the 2ABT. The Detect Cells feature was used to quantify cell positions in the anti-HA fluorescence channel (TRITC). Cells were identified based on size and intensity cutoffs calibrated to each brain. Cutoffs were determined by comparing manual annotations in a small area with NeuroInfo cell detection. Cell positions were then mapped to brain regions using the Allen Institute 2017 adult mouse common coordinate framework (CCF) atlas (25 μm/pixel) and counts per brain region were exported to a csv file. Mapped cell positions were exported from NeuroInfo into MATLAB, and a 3D Gaussian smoothing kernel (sigma=5 pixels) was applied before cross sections were isolated and overlayed onto the Allen Mouse Brain CCF Atlas to visualize WT- or DN-THR expression as a heatmap using the imoverlay package (Matt Smith, version 1.3.0.0).

#### Statistical methods

Sample sizes and statistical tests are listed either in the main text or figure captions. For pairwise comparisons in Figures 1, 2G, 5, 6, S1, S2, and S6, and multigroup comparisons in Figures S2D–S2F, normality of the groups was tested with Shapiro-Wilk test prior to statistical analyses with parametric or non-parametric tests. For pairwise comparisons in Figures 4, S3, and S5 and Table S3, normality of groups was not tested, and non-parametric tests were used. A description of the statistical methods used for differential gene expression (Figures 2 and S4) are described above in the snRNA-seq section. A description for statistical methods used for MoSeq analyses (Figure S3) are described within the analysis section above. Likelihood ratio tests were performed in R (4.2.2) on full versus reduced mixed effects models (MM) as follows: in Figures 1C and S1G–S1I, full models for energy expenditure, food consumption, and locomotion, temperature, probability of movement (movement bout was defined as at least 10 cm of movement in the 3 minute sampling period), and locomotion per bout used animal weight, light/dark period, treatment, experimental time, and the interaction of treatment and experimental time as fixed effects, and individual animals as random effects. Reduced models included all the same terms, but without an interaction term. For energy expenditure, temperature, and locomotion per bout we used linear MM (LMM). Food consumption, locomotion, and probability of movement were sparse datasets, so we used generalized linear MMs using zero-inflated negative binomial distributions or the Poisson distribution (probability of movement). For measures of L2/3 pyramidal neuron excitability (Figures S5A and S5B and caption), full models (LMM) used the injected current or log(current) and treatment as fixed effects, and individual cells as random effects. Reduced models lacked the treatment term. In Figure 5C, the full model (LMM) used experimental day, treatment, and the interaction of treatment and experimental day as fixed effects, and individual animals as random effects. The reduced model included all the same terms but without an interaction term. To understand after how many days of treatment reward rate was increased, we used LMMs with binary variables for each day of treatment as fixed effects and individual animals as random effects, including data only from animals treated with T3. Each reduced model had one of the experimental days dropped out. The full model in Figure 6C used experimental day, “genotype” (intracranial injections DN-THR or WT-THR), and the interaction of “genotype” and experimental day as fixed effects, and individual animals as random effects. The reduced model included all the same terms but without an interaction term. The models in Figures S6N and S6P used the same full and reduced models as Figure 6C, but with log(day) replacing experimental day as a fixed effect. All boxplots displayed in figures have central line indicating the median, box indicating inner quartiles (IQ: the 25th and 75th percentiles), and whiskers stretching to the largest/smallest data points that are at most 1.5x inter-quartile range (IQR).

## Supplementary Material

MMC4

MMC2

MMC3

MMC1

1

## Figures and Tables

**KEY RESOURCES TABLE:** 

REAGENT or RESOURCE Antibodies	SOURCE	IDENTIFIER

Antibodies		
Rat anti-HA	Roche	Cat# 1186742300; RRID: AB_390918
Chicken anti-GFP	Abcam	Cat# Ab13970; RRID: AB_300798
Rabbit anti-NeuN	Sigma Aldrich	Cat# ABN78; RRID: AB_10807945
Mouse anti-Robo3	R&D Systems	Cat# AF3155; RRID: AB_10644167
Mouse anti-GluN1	SySy	Cat# 114011; RRID: AB_887750
Goat anti-rat Alexa 555	ThermoFisher	Cat# A21434; RRID: AB_2535855
Goat anti-chicken Alexa 488	ThermoFisher	Cat# A11039; RRID: AB_2534096
Goat anti-mouse Alexa 647	ThermoFisher	Cat# A21235; RRID: AB_2535804
Streptavidin Alexa 647	ThermoFisher	Cat# S32357
Bacterial and virus strains		
scAAV9-hSyn-iCre-HA	Boston Children’s Hospital Viral Core	N/A
scAAV9-hSyn-ΔiCre-HA	Boston Children’s Hospital Viral Core	N/A
AAV9-hSyn-DN-THR	Boston Children’s Hospital Viral Core	N/A
AAV9-hSyn-WT-THR	Boston Children’s Hospital Viral Core	N/A
AAV9-SIO-nEF-DN-Thrb	Janelia Viral Tools	N/A
AAV9-SIO-nEF-WT-Thrb	Janelia Viral Tools	N/A
AAV9-hSyn-FLEX-CoChR-GFP	UNC GTC Vector Core	N/A
AAVretro-CAG-Cre	UNC GTC Vector Core	N/A
AAV9-hSyn-CoChR-GFP	UNC GTC Vector Core	N/A
pENN.AAV_PHPeB.hSyn.HI.eGFP-Cre.WPRE.SV40	Addgene	Addgene 105540
AAV9-S5E2-ChR2-mCherry	Addgene	Addgene 135634
Chemicals, peptides, and recombinant proteins		
3,3’,5-Triiodo-L-thyronine	Sigma-Aldrich	Cat# T6397
Sobetirome	Thomas Scanlan	N/A
Kolliphor	Sigma-Aldrich	Cat# C5135
NMP	Sigma-Aldrich	Cat# 328634
0.15% PTU Diet	Inotiv	Cat# TD.95125
Control Diet	Inotiv	Cat# TD.97350
Trizol	Life Technologies	Cat# 15596018
RIPA buffer	ThermoFisher	Cat# 89900
cOmplete, Mini, EDTA-free Protease Inhibitor Cocktail	Roche	Cat# 11836170001
Biocytin	Sigma Aldrich	Cat# B4261
Critical commercial assays		
Chromium Next GEM Single Cell 3’ Kit v3	10X Genomics	Cat#1000268
RNEasy Micro Kit	Qiagen	Cat#74004
SuperScript IV VILO Master Mix Kit with ezDNAse enzyme	ThermoFisher	Cat#11766050
RNAscope Multiplex Fluorescent Reagent Kit v1 (discontinued) and v2	Advanced Cell Diagnostics	Cat#323100
Deposited data		
snRNA-seq datasets	This paper	GEO: GSE271421
snRNA-seq datasets	This paper	https://cellxgene.cziscience.com/collections/c450e15d-321a-42d6-986b-11409d04896d
Moseq, electrophysiology, and 2ABT datasets	This paper	https://dataverse.harvard.edu/dataverse/2024_hochbaum_thyroid
Experimental models: Organisms/strains		
Mouse: C57BL/6J	The Jackson Laboratory	Cat# 664; RRID:IMSR_JAX:000664
Mouse: Robo3 ^fl/fl^: Robo3^tm1.1Ache^	Alain Chédotal^111^	MGI:4441335
Oligonucleotides		
TaqMan probe for *Hr*: (Mm00498963_m1)	ThermoFisher	Cat# 4331182
TaqMan probe for *Dio1*: (Mm00839358_m1)	ThermoFisher	Cat# 4331182
TaqMan probe for Ier5: (Mm01295615_s1)	ThermoFisher	Cat# 4331182
TaqMan probe for *Gapdh*: (Mm99999915_g1)	ThermoFisher	Cat# 4351370
TaqMan probe for *Cyp11a1*: (Mm00490735_m1)	ThermoFisher	Cat# 4331182
FISH probe for *Hr*	Advanced Cell Diagnostics	Cat# 883311
FISH probe for *Ier5*	Advanced Cell Diagnostics	Cat# 530401-C3
FISH probe for *Robo3*	Advanced Cell Diagnostics	Cat# 558811-C2
4-plex positive probe	Advanced Cell Diagnostics	Cat# 321811
4-plex negative control probe	Advanced Cell Diagnostics	Cat# 321831
Recombinant DNA		
DRH001_scAAV-hSyn-iCre-HA	This paper	Addgene 225086
DRH002_scAAV-hSyn-ΔiCre-HA	This paper	Addgene 225087
DRH015_ AAV-hSyn-WT-THR	This paper	Addgene 225088
DRH016_ AAV-hSyn-DN-THR	This paper	Addgene 225089
DRH031_ AAV-SIO-nEF-WT-THR	This paper	Addgene 225090
DRH032_ AAV-SIO-nEF-DN-THR	This paper	Addgene 225091
Software and algorithms		
Custom software	This paper	https://github.com/bernardosabatinilab/hochbaum_thyroid_2024
NeuroInfo	MBF Bioscience	https://www.mbfbioscience.com/products/neuroinfo/; RRID:SCR_017346
MoSeq behavior analysis pipeline	Lin et al.^54^	http://www.moseq4all.org/
ImageJ (Fiji)	Schindelin et al.^112^	https://imagej.net/; RRID: SCR_003070
CellRanger v.5 pipeline	10x Genomics	https://www.10xgenomics.com/support/software/cell-ranger/latest
Modified Seurat v.2 workflow	Gazestani et al.^113^	N/A
fGSEA package version 1.24.0	Korotkevich et al.^114^	N/A
MATLAB version R2021b	MathWorks	https://www.mathworks.com/products/matlab.html
R version 4.2.2	R Foundation	https://cran.r-project.org/bin/windows/base/old/4.2.2/
Python version 3.9	Python Software Foundation	https://www.python.org

## Highlights

- T3 directly activates cell-type-specific transcriptional programs in adult cortex
- T3-regulated gene programs (TRGPs) alter excitatory and inhibitory synaptic transmission
- TRGPs in frontal cortex drive changes in exploratory behaviors and decision-making
- TRGPs in cortex act to coordinate exploratory behaviors with whole-body metabolism
