# Supplementary material for: Thyroid hormone remodels cortex to coordinate body-wide metabolism and exploration
Source: Cell. Author manuscript; Available in PMC 2024 Oct 6. (PMC11455614; doi:10.1016/j.cell.2024.07.041)
Supplement: MMC3 [file NIHMS2019959-supplement-MMC3.pdf]

**Table S3. L2/3 pyramidal neuron electrophysiological properties across experiments. Related to Figure 4.**

Cell properties of L2/3 pyramidal neurons across conditions and experiments. Values are mean ± sem. Statistical comparisons are performed with Wilcoxon rank-sum test. *Note, cell properties are recorded at V= -70 mV except for the experiment with PV cell inputs labeled with channelrhodopsin. This experiment only measured IPSCs, and cell properties were recorded at V=0 mV.

| Experiment | $C_{membrane}$ (pF) | $C_{membrane}$ (p-value) | $R_{membrane}$ (MΩ) | $R_{membrane}$ (p-value) | $R_{series}$ (MΩ) | $R_{series}$ (p-value) | $I_{hold}$ (pA) | $I_{hold}$ (p-value) |
|---|---|---|---|---|---|---|---|---|
| Trans-hemispheric inputs (**Fig. 4**) | C: 218 ± 65<br>T3: 197 ± 55 | p=0.26 | C: 131 ± 57<br>T3: 130 ± 49 | p=0.98 | C: 10.3 ± 3.9<br>T3: 10.4 ± 3.5 | p=0.65 | C: -118 ± 89<br>T3: -106 ± 82 | p=0.67 |
| Trans-hemispheric inputs with optical PPR (**Fig. S5c**) | C: 218± 43<br>T3: 206 ± 55 | p=0.35 | C: 120 ± 52<br>T3: 135 ± 62 | p=0.63 | C: 14.6 ± 5.5<br>T3: 12.5 ± 2.4 | p=0.60 | C: -96 ± 75<br>T3: -83 ± 46 | p=0.91 |
| *PV cell inputs (**Fig. S5d-f**) | C: 163± 39<br>T3: 181 ± 50 | p=0.18 | C: 116 ± 58<br>T3: 87 ± 45 | p=0.07 | C: 13.0 ± 4.7<br>T3: 13.1 ± 4.5 | p=0.97 | C: 96 ± 44<br>T3: 118 ± 53 | p=0.09 |
| Trans-hemispheric inputs with WT-THRB (**Fig. S5g-i**) | C: 227 ± 52<br>T3: 214 ± 47 | p=0.33 | C: 124 ± 51<br>T3: 121 ± 43 | p=0.93 | C: 8.9 ± 2.6<br>T3: 9.1 ± 2.9 | p=0.73 | C: -75 ± 70<br>T3: -121 ± 94 | p=0.054 |
| Trans-hemispheric inputs with DN-THRB (**Fig. S5j-l**) | C: 233 ± 56<br>T3: 241 ± 60 | p=0.61 | C: 115 ± 39<br>T3: 118 ± 42 | p=0.89 | C: 7.8 ± 2.0<br>T3: 8.3 ± 2.7 | p=0.29 | C: -104 ± 103<br>T3: -120 ± 97 | p=0.31 |
| Trans-hemispheric inputs with ∆Cre, $Robo3^{+/+}$ (**Fig. S5m-o**) | C: 228 ± 43<br>T3: 227 ± 52 | p=0.87 | C: 114 ± 29<br>T3: 102 ± 42 | p=0.09 | C: 10.2 ± 3.2<br>T3: 9.9 ± 3.5 | p=0.61 | C: -130 ± 93<br>T3: -101 ± 92 | p=0.21 |
| Trans-hemispheric inputs with Cre, $Robo3^{-/-}$ (**Fig. S5p-r**) | C: 264 ± 77<br>T3: 240 ± 66 | p=0.31 | C: 94 ± 41<br>T3: 99 ± 36 | p=0.46 | C: 8.1 ± 2<br>T3: 9.8 ± 3.2 | p=0.06 | C: -97 ± 87<br>T3: -102 ± 119 | p=0.68 |
