# Supplementary material for: Thyroid hormone remodels cortex to coordinate body-wide metabolism and exploration
Source: Cell. Author manuscript; Available in PMC 2024 Oct 6. (PMC11455614; doi:10.1016/j.cell.2024.07.041)
Supplement: 1 [file NIHMS2019959-supplement-1.pdf]

CellPress
OPEN ACCESS

Supplemental figures

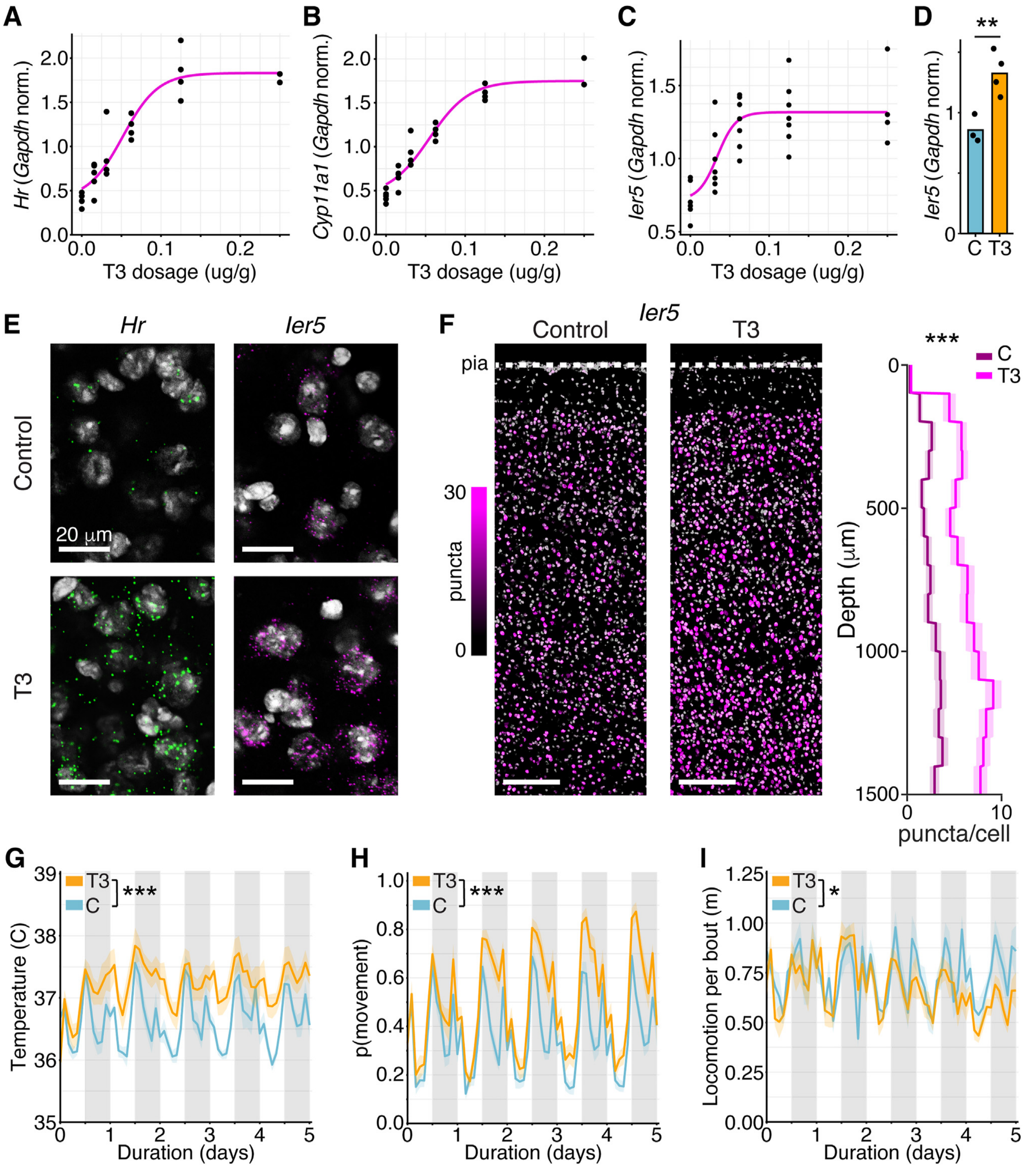

(legend on next page)

Figure S1. Characterization of T3 effects on cortical transcription and animal physiology, related to Figure 1

(A) *Hr* induction in frontal cortex as a function of T3 concentration. *Hr* expression saturates by ~0.125 μg/g. $n = 2$–4 mice per concentration. This dose was used for all subsequent experiments unless otherwise noted. Pink line fits the data to a sigmoidal curve.

(B) As in (A), for the TRG *Cyp11a1*. $n = 2$–4 mice per concentration.

(C) As in (A) and (B), for the TRG *Ier5*. $n = 4$–7 mice per concentration.

(D) *Ier5* is upregulated in frontal cortex 1 h after treatment with T3 relative to vehicle control (control: $n = 3$ mice; T3 treated: $n = 4$ mice; measured by qPCR; $p = 0.008$ Welch's t test).

(E) Images of FISH fluorescent puncta from tissues probed with *Hr* and *Ier5* from control or T3 conditions. Scale bars, 20 μm.

(F) Quantification of *Ier5* expression by FISH in M2 (left: control, middle: T3, nuclei pseudo-colored by number of detected *Ier5* puncta). Scale bars, 200 μm. Right: summary of *Ier5* expression as a function of depth. *Ier5* is upregulated by T3 across cortex ($p = 0$, Wilcoxon rank-sum test comparing treatment effect across entire cortical depth; control: $n = 5{,}494$ cells; T3 treated: $n = 6{,}061$; cortical slices from 2 mice per condition). Central line/shade: mean/95% confidence intervals.

(G) Mice in the home-cage indirect calorimetry system were implanted with telemetric temperature probes. Internal body temperature increased with T3 compared with controls over the experimental time course (linear mixed model, $p < 10^{-4}$, likelihood ratio test, $n = 7$ control, and $n = 7$ T3-treated mice). Central line/shade: mean/SEM.

(H) Mice in the home-cage indirect calorimetry system treated with T3 increased their probability of being active, defined as locomotion of more than 10 cm in the 3 min sampling period (generalized linear mixed model, $p < 10^{-4}$, likelihood ratio test, $n = 16$ control, and $n = 15$ T3-treated mice). Central line/shade: mean/SEM.

(I) Although more likely to be active, mice in the home-cage indirect calorimetry system treated with T3 decreased the amount they moved per bout of activity over the experimental time course (linear mixed model, $p = 0.04$, likelihood ratio test, $n = 16$ control, and $n = 15$ T3-treated mice). Central line/shade: mean/SEM.

For all panels: *$p < 0.05$, **$p < 0.01$, ***$p < 0.001$, black dots indicate data from single mice.

CellPress
OPEN ACCESS

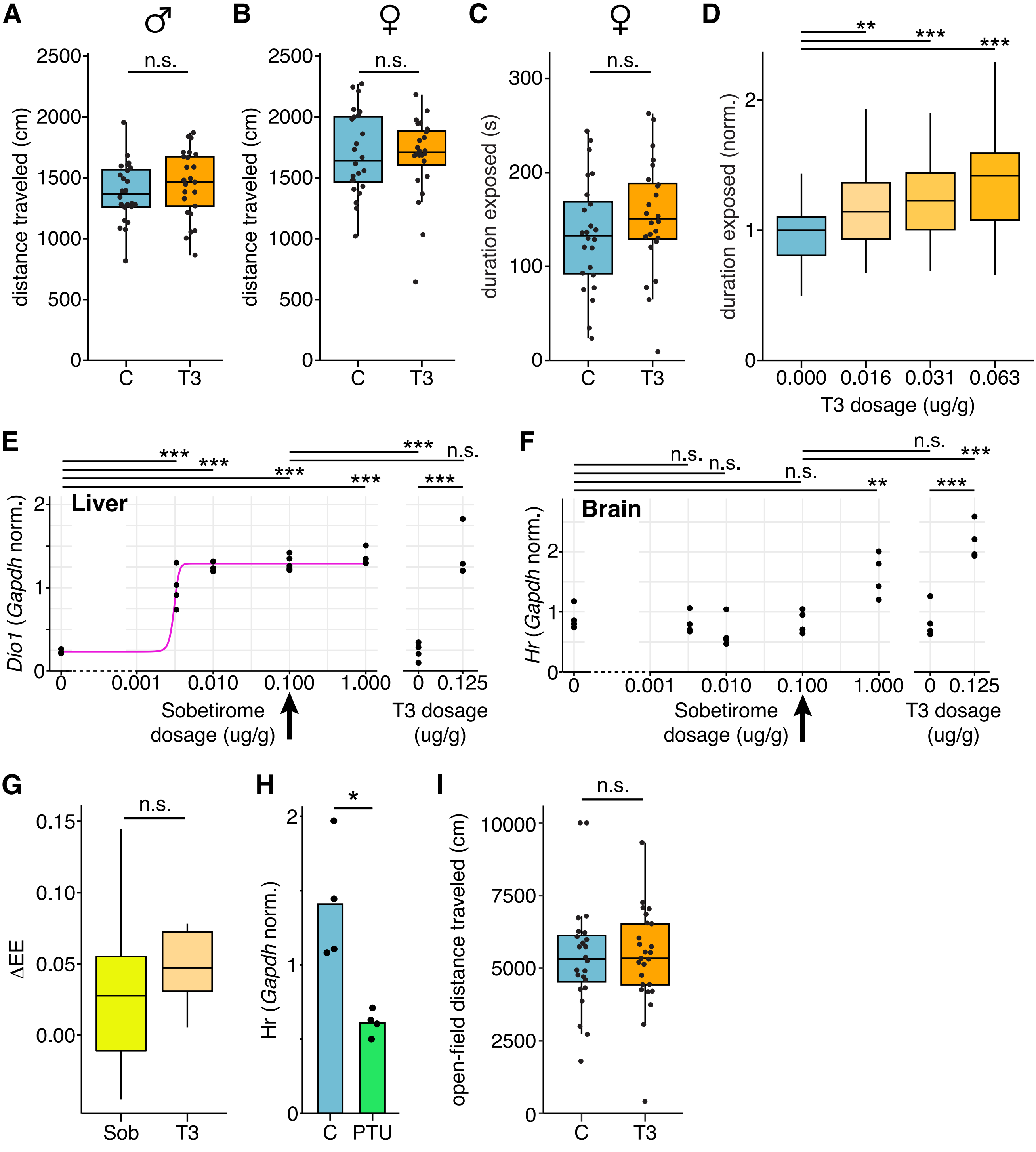

Figure S2. T3, sobetirome, and PTU affect transcription, physiology, and behavior, related to Figure 1

(A) In the LD assay, T3 treatment of male mice did not change overall distance traveled relative to controls ($p = 0.39$; $n = 24$ C, $n = 25$ T3-treated male mice), despite increasing the duration mice spent in the lit exposed portion of the LD assay (Figure 1E).

(B) T3 treatment of female mice did not change distance traveled in the LD assay relative to controls ($p = 0.98$; $n = 24$ C, $n = 24$ T3-treated female mice).

(C) T3 treatment of female mice did not change the time spent in the light-exposed zone of the LD assay ($p = 0.25$; $n = 24$ C, $n = 24$ T3-treated female mice).

(D) Duration male mice stay in the light-exposed zone of the LD assay as a function of T3 concentration (C: $n = 51$ mice; 0.016 μg/g T3: $n = 36$ mice; 0.031 μg/g T3: $n = 40$ mice; 0.063 μg/g T3: $n = 25$ mice). Experiments were carried out over many experimental rounds and were normalized to the median duration of each round's control cohort (4–8 mice) to account for round-to-round variability. There was a significant effect of treatment ($\chi^2(3) = 20.022$, $p = 2 \times 10^{-4}$,

(legend continued on next page)

Kruskal-Wallis's test) and duration exposed to light increased with T3 concentration (linear regression, $F = 17.66$ (1,150), $p < 10^{-4}$). Administration of T3 at each concentration significantly increased the duration of light exposure relative to control (0.016 μg/g T3: $p = 0.009$, 0.031 μg/g T3: $p = 8 \times 10^{-4}$, 0.063 μg/g T3: $p = 10^{-4}$; Dunn's post hoc test using Holm's adjustment for multiple comparisons).

(E) Induction of the TRG *Dio1* in the liver as a function of sobetirome concentration (left) or with T3 treatment (right). *Dio1* expression differed among treatments (one-way ANOVA, $F(6, 23) = 45.4$, $p < 10^{-4}$). Both T3 and every tested sobetirome concentration increased *Dio1* expression in the liver ($p < 10^{-4}$, Tukey HSD test) relative to controls. 0.1 μg/g sobetirome, used in behavioral experiments and marked with an arrow, induced *Dio1* equivalently to T3 treatment ($p = 0.95$, Tukey HSD test). Pink line fits the data to a sigmoidal curve.

(F) Induction of the TRG *Hr* in the brain (frontal cortex) as a function of sobetirome concentration (left) or with T3 treatment (right). *Hr* expression differed among treatments (one-way ANOVA, $F(6, 23) = 20.0$, $p < 10^{-4}$). T3 treatment ($p < 10^{-4}$, Tukey HSD test) increased *Hr* expression in the brain, as did 1 μg/g sobetirome ($p = 0.009$, Tukey HSD test), relative to controls. 0.1 μg/g sobetirome did not induce *Hr* in the brain (indicated by arrow; $p = 0.99$, Tukey HSD test), despite inducing *Dio1* in the liver (see E). Therefore, 0.1 μg/g sobetirome was chosen as the maximum concentration with peripheral TRG induction and without central TRG induction.

(G) Sobetirome (0.1 μg/g; $n = 17$ mice) vs. T3 (0.016 μg/g; $n = 14$ mice) treatment of mice resulted in a change in energy expenditure after 3.5 days of treatment that was similar between treatments ($p = 0.31$). The change in energy expenditure was calculated between treated mice and the average of control cohorts that were run simultaneously (sobetirome vehicle controls: $n = 18$ mice; T3 vehicle controls: $n = 13$ mice).

(H) PTU treatment over 3.5 weeks downregulated the TRG transcript *HR* in secondary motor cortex relative to controls (measured by qPCR; $n = 4$ mice per condition; $p = 0.03$).

(I) T3-treated ($n = 25$ mice) and control mice ($n = 24$ mice) were placed in a standard open-field assay, 4.5 days after treatment. Locomotion over the experimental time course was not affected by treatment ($p = 0.91$).

For all panels: n.s., not significant; $^{*}p < 0.05$, $^{**}p < 0.01$, $^{***}p < 0.001$, black dots indicate data from single mice. Boxplots central line: median, box: IQ, whiskers: data within 1.5× IQR. (A), (C), and (G)–(I) Statistical comparisons performed with Welch's t test; (B) statistical comparison performed with Wilcoxon rank-sum test.

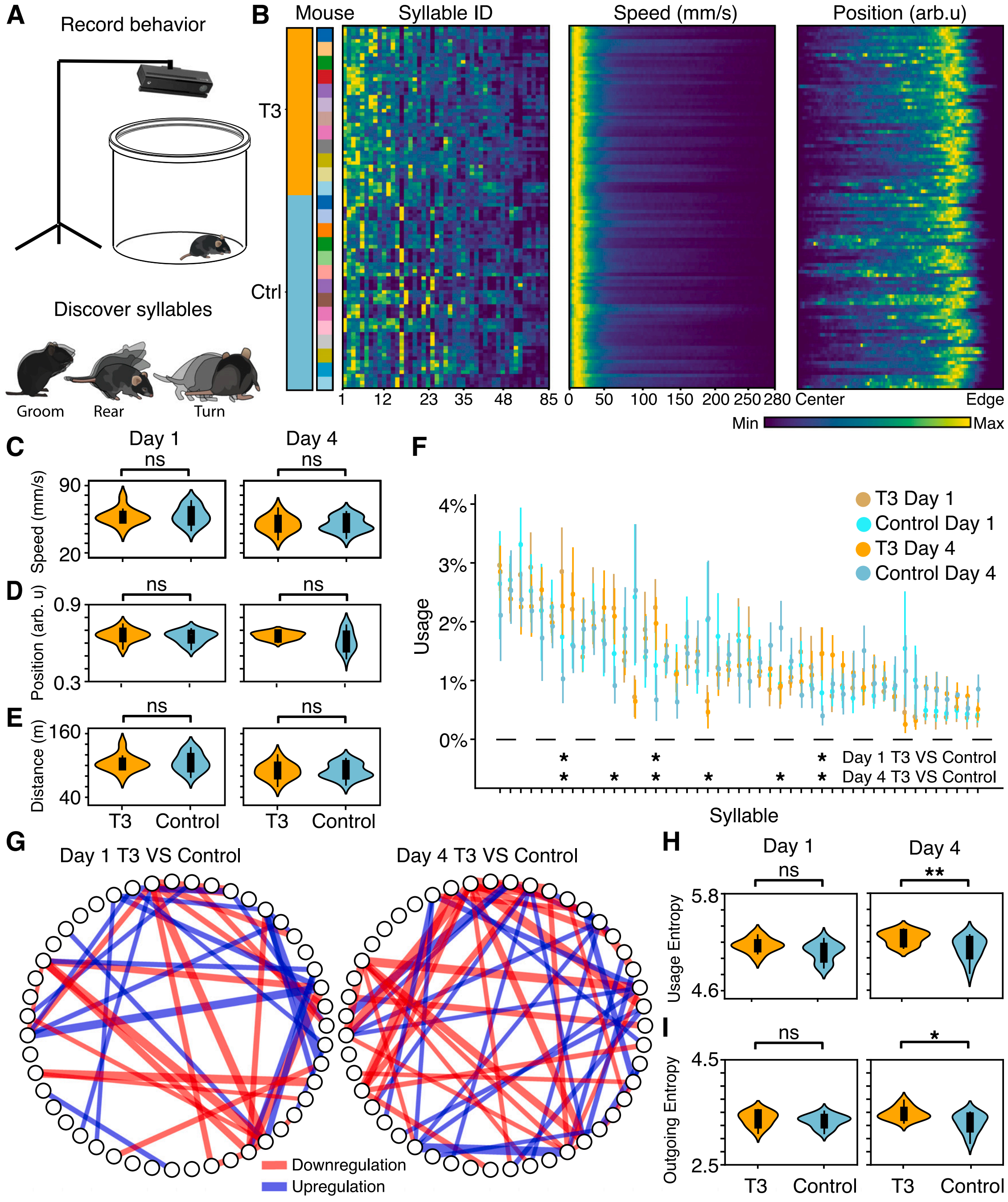

Figure S3. T3 treatment increases the diversity of spontaneous behavioral syllables and sequences, related to Figure 1

(A) Animals were habituated for 3 days to the behavioral room and an open-field arena. Animals were treated with T3 (n = 12) or control (n = 14) for 4 days. Each day, animals were placed in the arena, and their spontaneous 3D movements were recorded using a depth camera (top). MoSeq modeling segments the depth videos into behavior motifs, called syllables (3 examples are shown at the bottom).

(B) A heatmap showing MoSeq syllable ID (left), speed (center), and position (right, defined as the distance from arena center, normalized to the arena radius). Each row is one session, comprising one mouse recorded on one experimental day within the open-field arena. The heatmap is sorted by treatment, mouse, and experimental day, and each row in each panel is normalized to min/max value in the session.

(C) Mean speed of T3 and control cohorts in the first (day 1) and last (day 4) sessions displayed no difference with treatment. (Mann-Whitney U test, day 1: statistic = 78.0, p = 0.78; day 4: statistic = 80, p = 0.86).

(D) Mean position of T3 and control cohorts on day 1 and day 4 displayed no difference with treatment. (Mann-Whitney U test, day 1: statistic = 94.00, p = 0.63; day 4: statistic = 116.00, p = 0.11.)

(legend continued on next page)

(E) Total distance traveled of T3 and control cohorts on day 1 and day 4 displayed no difference with treatment. (Mann-Whitney U test, day 1: statistic 78.00, p = 0.78; day 4: statistic = 81.00, p = 0.90.)

(F) Mean syllable usage distribution for T3-treated and vehicle control cohorts on day 1 and day 4. Only syllables used more than 1% across all recordings are included. Asterisks represent differentially used syllables (FDR-adjusted p < 0.1, STAR Methods) between T3 and control on day 1 (top row) and day 4 (bottom row). Dots are means, error bars are 95% confidence intervals.

(G) Average transition probability differences across syllables between T3 and control cohorts on day 1 and day 4. The thickness of the edges scales with the magnitude of the differences. The increased number and thickness of edges on day 4 relative to day 1 indicate diverging statistics of syllable transitions between T3 and control cohorts.

(H) Usage entropy, a measure of behavioral variability—high entropy indicates the animals perform more types of behavior, low entropy indicates the animals perform a less diverse selection of behavior—of T3 and control cohorts were similar on day 1 of treatment (Mann-Whitney U test, statistic= 120.00, p =0.07); however, T3 usage entropy was significantly higher than control on day 4 (Mann-Whitney U test, statistic = 135, p = 0.01). Although the usage entropy of the control cohort did not change over the experimental time course (linear regression, F = 0.5041 (1, 54), p = 0.48), usage entropy of the T3 cohort increased over the experiment (linear regression, F = 6.854 (1, 46), p = 0.012).

(I) Syllable outgoing entropy, a measure of how variable an animal's behavior is conditional on its current syllable, was similar between T3 and control cohorts on day 1 (Mann-Whitney U test, statistic = 103.00, p = 0.34) but was elevated in the T3-treated cohort on day 4 (Mann-Whitney U test, statistic = 126.00, p = 0.03). For all panels with boxplots, box: IQ, whiskers: data within 1.5× IQR.

CellPress
OPEN ACCESS

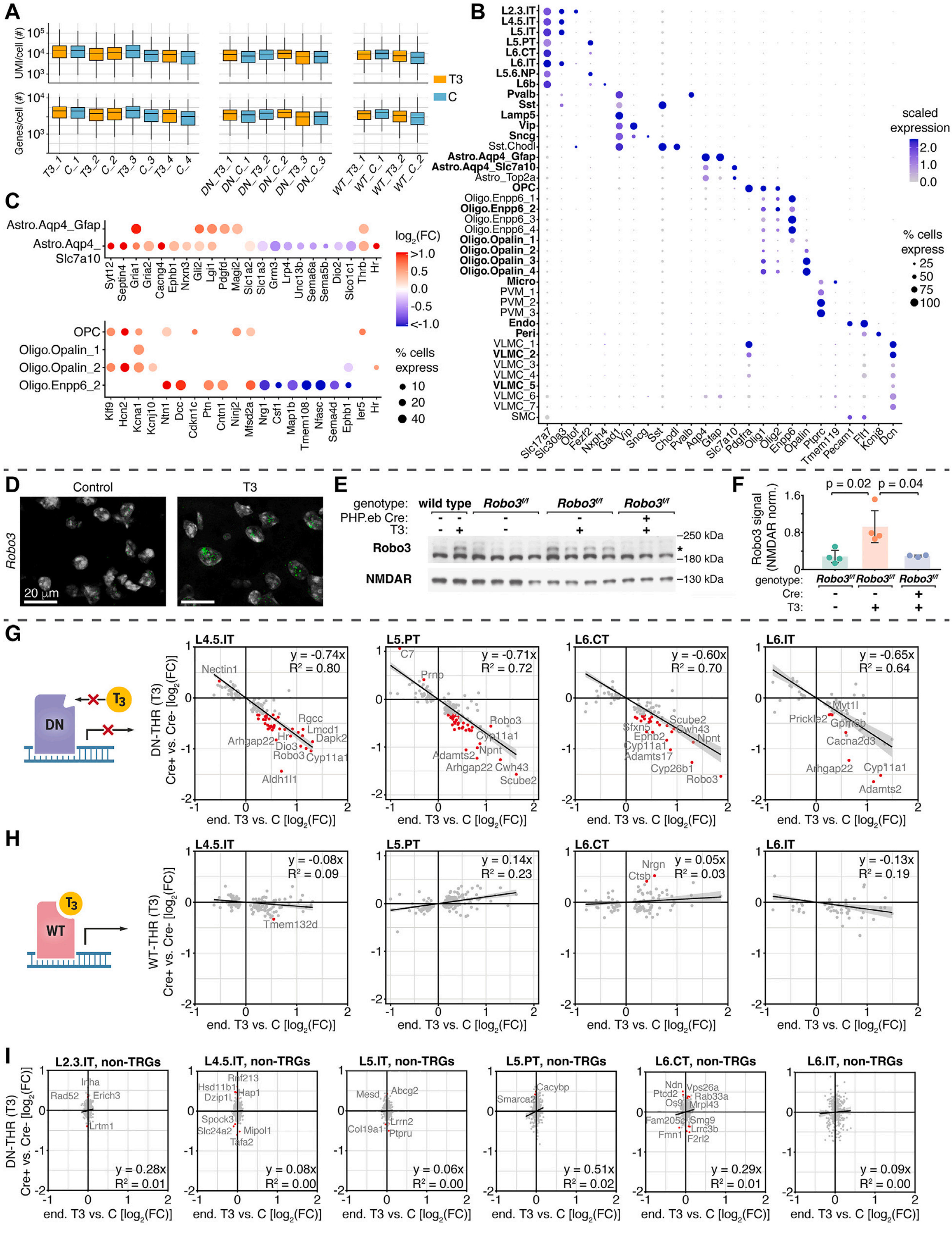

(legend on next page)

Figure S4. Local T3-dependent transcriptional programs in M2 identified by snRNA-seq, related to Figures 2 and 3

(A) Top: boxplot showing the distribution of the number of unique molecular identifiers (nUMIs) detected in each nucleus over all samples, after quality control filtering. Samples (T3 in orange, vehicle controls in cyan) showed consistent UMI distributions. Samples T3_1-4 and C_1-4 are from mice treated with either T3 or vehicle control solutions and reported in Figure 2. Samples DN_T3_1-3, DN_C_1-3, WT_T3_1-2, and WT_C_1-2 refer to animals that received intracranial injections of AAVs encoding Cre along with a Cre-dependent dominant-negative (DN) or wild-type (WT) thyroid receptor, and treatment with T3 or vehicle. A subset of these samples (DN-THR, T3: 47,876 cells, WT-THR, T3: 45,076 cells) are reported in Figure 3. Bottom: as in top but for the number of genes (nGenes) expressed in each cell. Samples showed consistent gene expression distributions. Central line: median, box: IQ, whiskers: data within 1.5× IQR.

(B) Marker gene dot plot. After clustering into broad cell classes, cells were mapped onto previously defined cell types from a single-cell atlas of motor cortex.[55] Mapped cell types displayed appropriate expression of marker genes. Cell types in bold had sufficient cell counts to perform analysis of differential gene expression between T3 and C conditions. Dot color indicates the marker gene's scaled (*Z* score) expression, dot size indicates the percentage of cells in each cell type that expressed one or more transcripts.

(C) Top: dot plot highlighting TRGs that drive GSEA enriched pathways in astrocytes and are associated with astrocyte regulation and assembly of synapses. These include astrocyte-expressed genes such as *Septin4*, *Slc1a2*, and *Slc1a3*, which encode proteins implicated in glutamate clearance,[121,122] and *Lrp4*, which encodes an astrocytic modulator of glutamatergic synaptic release probability.[123] Additional TRGs are included that indicate a homeostatic response to T3 (downregulation of *Slco1a1* and *Dio2*, upregulation of *Thrb*).

Bottom: dot plot highlighting TRGs that drive GSEA enriched pathways in OPCs and oligodendrocytes and are associated with OPC and oligodendrocyte differentiation, maturation, and myelination. These include genes encoding transcription factors such as *Klf9* known to promote oligodendrocyte differentiation and myelin regeneration,[60] and ion channels such as *Hcn2*, which regulates myelin sheath length,[124] along with secreted factors such as *Ntn1*, which is implicated in oligodendrocyte maturation[125] and is a ligand of the Robo3/DCC complex.[63] Dot color: fold-change in expression between the control and T3 conditions. Size: percentage of cells in each cell type that expressed one or more transcripts of the given gene in the T3 condition.

(D) FISH images of fluorescent puncta from tissues probed with *Robo3* from control or T3 conditions. Scale bars, 20 μm.

(E) Western blot from frontal cortex of wild-type and $Robo3^{fl/fl}$ animals with and without T3 treatment and/or Cre delivery. Staining was performed with antibodies for Robo3 and the Glun1 subunit of the NMDA-type glutamate receptor to normalize for neuronal content in each sample. Robo3 antibody staining resulted in two bands, one of which increased in intensity with T3 treatment. We delivered Cre to excise Robo3 and prevent its induction with T3. Only the top band (*), whose intensity was altered by T3, was eliminated, demonstrating that this band is specific to Robo3 and that the other band is non-specific.

(F) Robo3 protein levels increased with T3 treatment ($p = 0.02$) and were occluded by Cre excision of Robo3 ($p = 0.04$). Bars/error bars: mean/SD, and *p* values computed with two-tailed t tests.

(G) Plots for subtypes of glutamatergic projection neurons (left to right: L4.5.IT, L5.PT, L6.CT, L6.IT; L2.3.IT, L5.IT displayed in Figure 3) showing on the y axes the $\log_2$(fold-change) of TRGs between Cre+ (DN-THR expressing) cells and Cre− (lacking DN-THR) cells, after T3 treatment. The x axes show the $\log_2$(fold-change) of TRGs for each subtype between the T3 and control conditions from the original dataset (Figure 2). Red dots highlight TRGs whose expression was significantly disrupted by 25% or more due to DN-THR (FDR-adjusted $p < 0.05$, and fractional change in expression of at least ±25%). Linear regression fits to the data are overlaid; gray shading indicates a 95% confidence interval. Fit equation, and R^2 value are displayed on the upper right.

(H) As in (G), but for WT-THR expressing tissue. The y axes show the $\log_2$(fold-change) of TRGs between Cre+ (WT-THR expressing) cells and Cre− (lacking WT-THR) cells for each subtype, after T3 treatment.

(I) Plots for subtypes of glutamatergic projection neurons (left to right: L2.3.IT, L4.5.IT, L5.IT, L5.PT, L6.CT, L6.IT) showing on the y axes the $\log_2$(fold-change) of genes not regulated by T3 (non-TRGs) between Cre+ (DN-THR expressing) cells and Cre− (lacking DN-THR) cells, after T3 treatment. The x axes show the $\log_2$(fold-change) of non-TRGs for each subtype between the T3 and control conditions from the original dataset (Figure 2). Red dots indicate non-TRGs whose expression was significantly disrupted by 25% or more due to DN-THR (FDR-adjusted $p < 0.05$, and fractional change in expression of at least ±25%). Linear regression fits to the data are overlaid, and gray shading indicates a 95% confidence interval. Fit equation and R^2 value are displayed on the lower right.

CellPress
OPEN ACCESS

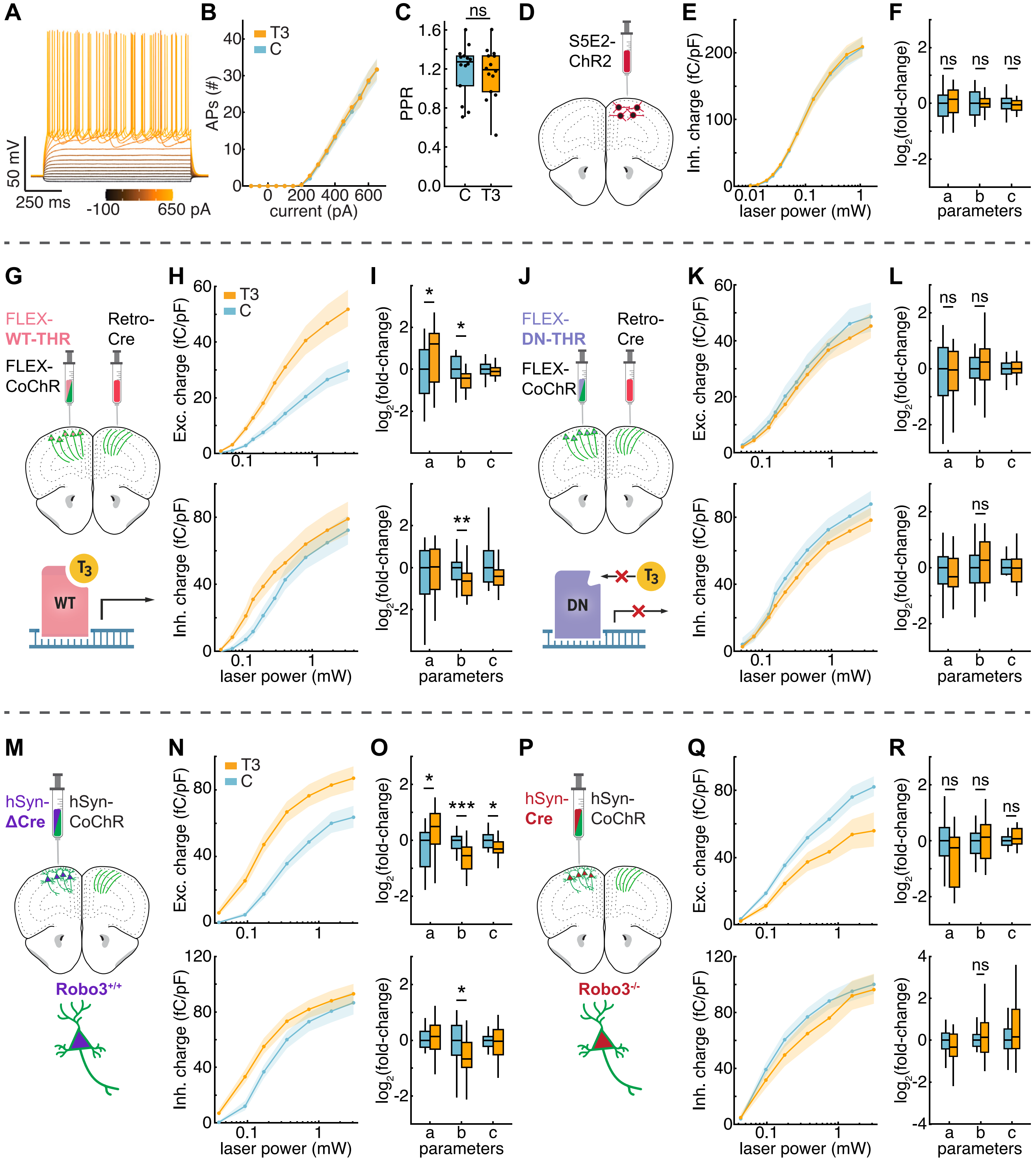

Figure S5. T3-dependent presynaptic gene regulation modifies cell-type-specific synaptic connectivity, related to Figure 4

(A) Example current-clamp whole-cell recording. Recordings were obtained from L2/3 pyramidal neurons in M2 in acute brain slices from T3 (n = 36 cells, 7 animals) or vehicle control (n = 30 cells, 7 animals) treated animals. Current steps (1 s) were injected from −100 to 650 pA. Traces are color coded by the amplitude of the injected current step.

(B) Input-output curve of injected current (I) vs. number of generated action potentials (APs). There was no observed effect on the generation of APs due to T3 treatment (p = 0.85). Additional metrics of excitability were not sensitive to T3 treatment, including plateau potential (p = 0.17), and the half-width (p = 0.73), peak voltage (p = 0.85), and maximum rate of change in voltage (dV/dt, p = 0.43) for the first generated AP at each injected current. Experiments were repeated using

(legend continued on next page)

alternate divalent cation concentrations (1 mM Ca^{2+}, 2 mM Mg^{2+}; control: $n = 10$ cells, 2 animals, T3-treated: $n = 11$ cells, 2 animals) and did not reveal any modulation by T3 (I vs. AP: $p = 0.99$; I vs. plateau: $p = 0.76$; I vs. half-width: $p = 0.43$; I vs. peak: $p = 0.70$; I vs. max dV/dt: $p = 0.55$). Dots/shading: mean/bootstrapped SEM.

(C) Summary of optical paired pulse ratio (PPR; 473 nm laser stimuli of CoChR-GFP expressing trans-hemispheric axons separated by 50 ms) recordings from recipient L2/3 pyramidal neuron. There was no significant difference in PPR with treatment ($p = 0.73$; T3-treated: $n = 14$ cells, 6 mice, orange; vehicle-treated: $n = 14$ cells, 5 mice, blue). Laser power was tuned to yield similarly sized EPSCs (~100–500 pA) from cells in acute brain slices from control and T3-treated mice ($p = 0.60$). There was no correlation between PPR and initial EPSC size (linear regression, F = 0.465 (1, 26), $p = 0.50$). Each dot represents data from a single recorded neuron.

(D) An AAV encoding ChR2-mCherry driven by a designed regulatory element (S5E2) resulting in expression in parvalbumin (PV) interneurons in cortex[73] was delivered to the upper layers of M2. Acute brain slices were prepared, and whole-cell recordings were obtained from L2/3 pyramidal neurons within the field of ChR2-expressing PV interneurons within M2. Monosynaptic inhibitory currents triggered by whole-field light stimulation across a range of laser intensities were recorded.

(E) Normalized post-synaptic inhibitory charge as a function of laser stimulus power (T3-treated: $n = 30$ neurons, 8 mice, orange; vehicle-treated: $n = 33$ neurons, 8 mice, blue). Dots/shading: mean/bootsrapped SEM.

(F) Boxplot of changes in each sigmoid parameter (from single-cell fits of inhibitory charges vs. laser power curves) relative to the median control value. T3 treatment had no effect on the saturation amplitude (a; $p = 0.62$), the power to half-maximum (b; $p = 0.42$), or the slope of the response curve (c; $p = 0.55$).

(G) Top: AAVs encoding a Cre-dependent CoChR (FLEX-CoChR) and a Cre-dependent WT-THR (FLEX-WT-THR) were co-delivered to the upper layers of M2. A retrograde AAV encoding Cre was delivered to the contralateral hemisphere, resulting in CoChR and nuclear WT-THR expression selectively in neurons sending projections to contralateral M2. Whole-cell recordings were obtained from L2/3 pyramidal neurons within the field of CoChR-expressing axons in contralateral M2. Monosynaptic excitatory currents and di-synaptic inhibitory currents triggered by whole-field light stimulation across a range of laser intensities were recorded. Bottom: cartoon of WT-THR expression.

(H) Top: normalized post-synaptic excitatory charge as a function of laser stimulus power from experiments with WT-THR expression in the presynaptic neurons (T3-treated: $n = 27$ neurons, 10 mice, orange; vehicle-treated: $n = 30$ neurons, 9 mice, blue). Bottom: as in top, but for normalized inhibitory charge. Dots/shading: mean/bootsrapped SEM.

(I) Top: boxplot of changes in each sigmoid parameter (from single-cell fits of excitatory charges vs. laser power curves) relative to the median control value for experiments with WT-THR expression in the presynaptic neurons. The saturation amplitude (a) was significantly increased by T3-treatment ($p = 0.026$), and the power to half-maximum (b) was significantly decreased by T3-treatment ($p = 0.014$). The slope of the response curve (c) was not effected by T3-treatment ($p = 0.22$). Bottom: as in top, but for single-cell fits of inhibitory charge vs. laser power curves. Power to half-maximum (b) was significantly decreased by T3 treatment ($p = 0.004$). Amplitude (a; $p = 0.59$) and slope (c; $p = 0.08$) were unaltered by T3-treatment.

(J) Top: as in (G), but for experiments that replaced FLEX-WT-THR with FLEX-DN-THR, an AAV encoding a Cre-dependent DN-THR. Bottom: cartoon of DN-THR expression, which perturbs T3-dependent gene transcription.

(K) As in (H), but for experiments with DN-THR expressing in presynaptic neurons (T3-treated: $n = 31$ neurons, 8 mice, orange; vehicle-treated: $n = 30$ neurons, 8 mice, blue).

(L) As in (I), but for experiments with DN-THR expressing in presynaptic neurons. Top: for fits of excitatory charge vs. laser power, the saturation amplitude (a; $p = 0.96$), power to half-maximum (b; $p = 0.52$), and slope (c; $p = 0.81$) were not altered by T3-treatment. Bottom: for fits of inhibitory charge vs. laser power; amplitude (a; $p = 0.42$) the power to half-maximum (b; $p = 0.36$), and slope (c; $p = 0.70$) were not altered by T3 treatment.

(M) Top: AAVs encoding an inactive control ΔCre (incapable of catalyzing recombination, hSyn-ΔCre) and a Cre-independent CoChR-GFP (hSyn-CoChR), both driven by neuronal specific promoter hSyn, were co-delivered to the upper layers of M2. Whole-cell recordings were obtained from L2/3 pyramidal neurons within the field of CoChR-expressing axons in contralateral M2. Monosynaptic excitatory currents and di-synaptic inhibitory currents triggered by whole-field light stimulation across a range of laser intensities were recorded. Bottom: cartoon of a CoChR-expressing cortico-cortical projecting IT neuron also expressing ΔCre, leaving *Robo3* expression intact.

(N) Top: normalized post-synaptic excitatory charge as a function of laser stimulus power from experiments with presynaptic ΔCre and CoChR-GFP expression (T3-treated: $n = 33$ neurons, 7 mice, orange; vehicle-treated: $n = 17$ neurons, 4 mice, blue). Bottom: as in top, but for normalized inhibitory charge. Dots/shading: mean/bootsrapped SEM.

(O) Top: boxplot of changes in each sigmoid parameter relative to the median control value for fits of excitatory charge vs. laser power in experiments including ΔCre expression in the presynaptic neurons. These experiments recapitulated previously observed changes due to T3. The saturation amplitude (a) was significantly increased by T3 treatment ($p = 0.017$), and the power to half-maximum (b) was significantly decreased by T3 treatment ($p < 0.001$). The slope of the sigmoid (c) was also significantly decreased by T3 treatment ($p = 0.01$) consistent with the trend observed in previous experiments (top of I, Figure 4E). Bottom: as in top, but for single-cell fits of inhibitory charge vs. laser power curves. Experiments with ΔCre expression in the presynaptic neurons recapitulated previous findings of T3-dependent changes to di-synaptic inhibition. The power to half-maximum was significantly decreased by T3 treatment ($p = 0.013$). Amplitude (a; $p = 0.41$) and slope (c; $p = 0.70$) were not altered by T3 treatment.

(P) Top: as in (M), but for experiments that replaced the inactive control ΔCre with a functional Cre (hSyn-Cre), leading to the loss of Robo3 expression (Figures S4E and S4F). Bottom: cartoon of a cortico-cortical projecting IT neuron expressing Cre, leading to the loss of *Robo3* expression.

(Q) As in (N), but for experiments with Cre expressing in presynaptic neurons (T3-treated: $n = 17$ neurons, 5 mice, orange, vehicle-treated: $n = 33$ neurons, 7 mice, blue).

(R) As in (O), but for experiments with Cre expressing in presynaptic neurons. Top: for fits of excitatory charge vs. laser power, the saturation amplitude (a) was no longer altered by T3-treatment ($p = 0.06$), the power to half-maximum (b) was not altered by T3 treatment ($p = 0.95$), and the slope was not altered by T3 treatment ($p = 0.57$). Bottom: for fits of inhibitory charge vs. laser power, the power to half-maximum was no longer altered by T3-treatment ($p = 0.76$). Amplitude (a; $p = 0.30$) and slope (c; $p = 0.41$) remained unaltered by T3 treatment.

For (B), *p* values were calculated by likelihood ratio tests comparing linear mixed-effects models (LMMs) including the treatment condition vs. LMMs that did not. For all other panels, statistical comparisons were performed with Wilcoxon rank-sum tests. n.s., not significant; *$p < 0.05$, **$p < 0.01$, ***$p < 0.001$. For all panels with boxplots, central line: median, box: IQ, whiskers: data within 1.5× IQR.

CellPress
OPEN ACCESS

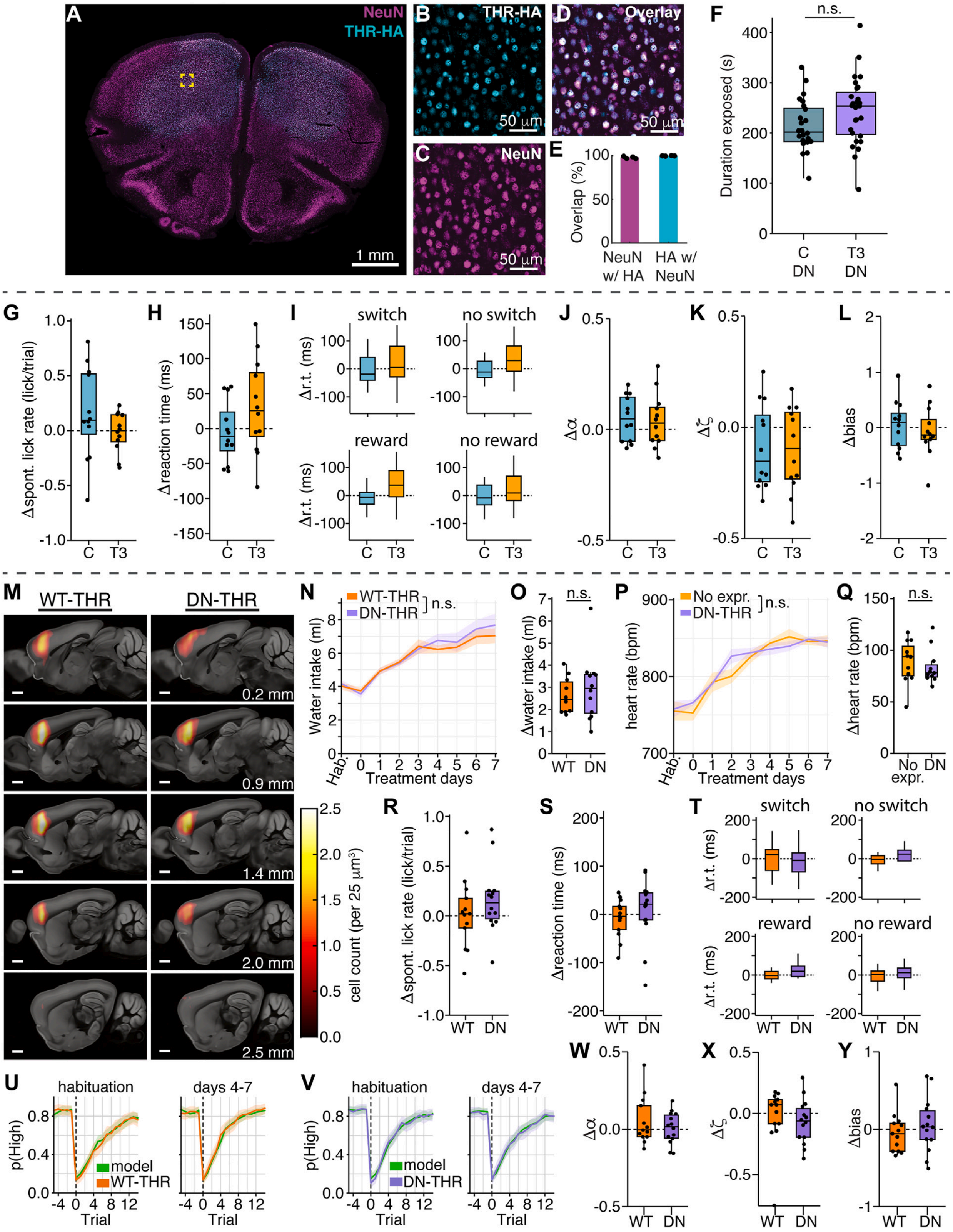

(legend on next page)

Figure S6. Behavioral effects mediated by T3-dependent transcriptional programs in frontal cortex neurons, related to Figures 5 and 6

(A) Low-magnification image showing example THR expression driven by neuronal specific promoter hSynapsin-1 in frontal cortex (~2.5 mm anterior to bregma) after intracranial injection of an AAV encoding WT-THR. Immunohistochemistry was performed with antibodies for HA (the C-terminal tag for WT- and DN-THR constructs, cyan) and neuronal peri-nuclei (NeuN, magenta). Scale bar, 1 mm.

(B) High-magnification image of HA staining from the yellow region highlighted in (A). Scale bar, 50 μm.

(C) As in (B) with NeuN staining. Scale bar, 50 μm.

(D) Overlay of images from (B) and (C). Scale bar, 50 μm.

(E) Quantification of HA and NeuN overlap. HA was present in 97.78% (1,274/1,302 cells) of NeuN+ peri-nuclei, and 99.72% (1,522/1,527 cells) of HA+ nuclei had NeuN expression. Bars indicate mean, black dots indicate individual samples (2 field of views from 2 animals, 4 samples total). Each quantification was validated near the center of the injection site.

(F) Mice expressing DN-THR in frontal cortex neurons were treated with T3 (purple, $n = 27$) or vehicle control (dark blue, $n = 24$) for 3.5 days and assessed in the LD assay as before (Figure 1E, left). In these mice, T3 treatment did not significantly increase time spent in the light-exposed zone of the LD assay ($p = 0.08$, Welch's t test).

(G) Change in the spontaneous lick rate (licks/trial) between the habituation period and days 4–7 of treatment for each experimental cohort (control cohort, blue; T3 cohort, orange). The spontaneous lick rate is defined as the average number of licks during the un-cued no-lick period that precedes the auditory tone for each trial. To exclude lick bouts due to consumption of a reward, only trials in which the previous trial was unrewarded are included in this analysis. Neither cohort had a significant change in spontaneous lick rate (control cohort, $p = 0.23$; T3 cohort, $p = 0.77$ paired t test).

(H) Change in the reaction time (ms) of the selection lick after the auditory tone (measure as the time between tone onset and lick contact with the spout) between the habituation period and days 4–7 of treatment for each experimental cohort. Neither cohort had a significant change in reaction time (control cohort, $p = 0.70$; T3 cohort, $p = 0.14$, paired t test).

(I) Change in the reaction time (ms) between the habituation period and days 4–7 of treatment for each experimental cohort conditional on whether the trial resulted in a switch in motor action (top row) or whether the trial followed a rewarded or unrewarded trial (bottom row). Neither cohort had a significant change in any of the conditional reaction times (switch: $p = 0.96$ for C, $p = 0.64$ for T3; no switch: $p = 0.71$ for C, $p = 0.12$ for T3; previous trial rewarded: $p = 0.63$ for C, $p = 0.12$ for T3; previous trial unrewarded: $p = 0.77$ for C, $p = 0.17$ for T3; paired t tests).

(J) Change in the learning rate parameter α (Q-learning model) between the habituation period and days 4–7 of treatment for each experimental cohort (control cohort, blue; T3 cohort, orange). Neither cohort had a significant change in α (control: $p = 0.11$; T3: $p = 0.28$, paired t test).

(K) Change in the forgetting rate parameter ζ (Q-learning model) between the habituation period and days 4–7 of treatment for each experimental cohort (control cohort, blue; T3 cohort, orange). Neither cohort had a significant change in ζ (control: $p = 0.14$; T3: $p = 0.10$, paired t test).

(L) Change in the bias parameter "b" (Q-learning model) between the habituation period and days 4–7 of treatment for each experimental cohort (control cohort, blue; T3 cohort, orange). Neither cohort had a significant change in "b" (control: $p = 0.70$; T3: $p = 0.64$, paired t test).

(M) Sagittal sections characterizing average WT-THR (left, $n = 12$ mice) and DN-THR expression (right, $n = 10$ mice). A full list of counts per brain region are in Table S4. Medial/lateral axis measures relative to the midline are listed in the lower right. Scale bars, 1 mm.

(N) Animals expressing WT-THR (dark orange, $n = 10$) or DN-THR (purple, $n = 12$) in frontal cortex were treated as in the 2ABT: animals were habituated with vehicle injections and then treated with T3 for 8 days. There was no effect of DN-THR expression on water intake over the experimental time course ($p = 0.38$, likelihood ratio test). Similarly, there was no effect of DN-THR expression on water intake normalized to mouse weight ($p = 0.38$, likelihood ratio test) Lines/shading: mean/SEM.

(O) Change in water intake between the habituation period and treatment days 4–7 did not differ between WT-THR and DN-THR cohorts ($p = 0.52$, Welch's t test). Similarly, change in weight-normalized water intake between the habituation period and days 4–7 of treatment did not differ between cohorts ($p = 0.49$, Welch's t test).

(P) Heart rate was recorded with a pulse oximeter in DN-THR expressing animals (purple, $n = 11$) and age-matched controls with no expression in frontal cortex (orange, $n = 11$). There was no effect of DN-THR expression on heart rate over the experimental time course ($p = 0.85$, likelihood ratio test). Lines/shading: mean/SEM.

(Q) Change in heart rate between the habituation period and days 4–7 of treatment did not differ between the no expression and DN-THR cohorts ($p = 0.37$, Wilcoxon rank-sum test).

(R) As in (G), but for the WT-THR expressing (dark orange, $n = 13$) and DN-THR expressing (purple, $n = 14$) cohorts. Neither cohort had a significant change in spontaneous lick rate (WT-THR: $p = 0.82$; DN-THR: $p = 0.10$, paired t test).

(S) As in (H), but for WT-/DN-THR cohorts. Neither cohort had a significant change in reaction time between habituation and days 4–7 of treatment (WT-THR cohort, $p = 0.46$; DN-THR cohort, $p = 0.65$, paired t test).

(T) As in (I), but for WT-/DN-THR cohorts. Neither cohort had a significant change in any of the conditional reaction times between habituation and days 4–7 of treatment (switch: $p = 0.88$ for WT-THR, $p = 0.66$ for DN-THR; no switch: $p = 0.34$ for WT-THR, $p = 0.27$ for DN-THR; previous trial rewarded: $p = 0.44$ for WT-THR, $p = 0.47$ for DN-THR; previous trial unrewarded: $p = 0.77$ for WT-THR, $p = 0.58$ for DN-THR; paired t tests or Wilcoxon signed-rank tests dependent on whether distributions were significantly non-Gaussian).

(U) Q-learning model fits of the probability of selecting the highly rewarding spout, p(High). Data from the habituation period (left) and days 4–7 (right). Orange lines: mean probability from the WT-THR mouse data; green lines: model prediction. Shading: 95% confidence intervals. The model fit the data well for both epochs (spout-choice prediction accuracy on held-out data during habituation for WT-THR cohort: 0.88 ± 0.03, mean $\pm$ SD; and during days 4–7 of treatment for WT-THR cohort: 0.87 ± 0.03; comparison between epochs: $p = 0.21$, paired t test).

(V) As in (U), but for the DN-THR mouse data. The model fit the data well for both epochs (spout-choice prediction accuracy on held-out data during habituation for DN-THR cohort: 0.87 ± 0.04; and during days 4–7 of treatment for DN-THR cohort: 0.84 ± 0.05; comparison between epochs: $p = 0.13$, paired t test).

(W) Change in the learning rate parameter α (Q-learning model) between the habituation period and days 4–7 of treatment for each experimental cohort. Neither cohort had a significant change in α (WT-THR: $p = 0.23$; DN-THR: $p = 0.79$, paired t test).

(X) Change in the forgetting rate parameter ζ (Q-learning model) between the habituation period and days 4–7 of treatment for each experimental cohort. Neither cohort had a significant change in ζ (WT-THR: $p = 0.79$, Wilcoxon signed-rank test; DN-THR: $p = 0.18$, paired t test).

(Y) Change in the bias parameter "b" (Q-learning model) between the habituation period and days 4–7 of treatment for each experimental cohort. Neither cohort had a significant change in "b" (WT-THR: $p = 0.52$; DN-THR: $p = 0.59$, paired t test).

For (G)–(L), $n = 12$ mice for each treatment condition (T3 or control). For (R)–(Y), WT-THR cohort: $n = 13$ mice, DN-THR cohort: $n = 14$ mice. For (F)–(H), (J)–(L), (O), (Q)–(S), and (W)–(Y), black dots represent data from individual mice. n.s., not statistically significant. For all boxplots, central line: median, box: IQ, whiskers: data within 1.5× IQR.
